# STIM1 as an Early Predictive Biomarker for Acute Respiratory Distress Syndrome (ARDS) and Its Potential Mechanisms

**DOI:** 10.1155/humu/9013000

**Published:** 2026-04-17

**Authors:** Shuping Deng, Qi Wu, Dan Zhou, Lijie Wang, Fangyuan Nan, Fang Dong, Jianguo Li

**Affiliations:** ^1^ Department of Intensive Care Unit, ZhongNan Hospital Affiliated to Wuhan University, Wuhan, Wu-Chang District, China; ^2^ Department of Scientific Research and Education, WuHan Third Hospital, Wuhan, Wu-Chang District, China, whsyy.net; ^3^ Department of Intensive Care Unit, WuHan Third Hospital, Wuhan, Wu-Chang District, China, whsyy.net; ^4^ Department of Thoracic surgery, The First People′s Hospital of Jiangxia District, Wuhan, Jiangxia District, China

**Keywords:** ARDS, inflammation, MAPK pathways, STIM1

## Abstract

Acute respiratory distress syndrome (ARDS) mainly results in severe respiratory failure and significant morbidity. This study decisively investigated the effectiveness and clinical importance of stromal interaction molecule 1 (STIM1) mRNA as an early predictive biomarker for the outcomes and severity of ARDS cases. A total of 72 mechanically ventilated patients were included in the study, consisting of 51 with ARDS and 21 without. The STIM1 mRNA levels in blood and bronchoalveolar lavage fluid (BALF) from these cases were assessed by quantitative reverse transcription polymerase chain reaction (RT‐qPCR). Additionally, lung tissues were collected from six patients (three with ARDS and three without) for hematoxylin–eosin staining, immunohistochemistry, and Western blot analysis. A predictive nomogram was constructed using STIM1 mRNA level and assessed for its ability to predict the severity and mortality of ARDS. A higher STIM1 mRNA level was associated with increased ARDS severity and patient mortality. Thus, STIM1 may serve as a novel biomarker for the early prediction of clinical outcomes and disease status of ARDS cases. Additionally, analysis of the GSE database revealed the MAPK signaling pathway as a key mechanism underlying the function of STIM1 in ARDS.

## 1. Introduction

Acute lung injury (ALI) and acute respiratory distress syndrome (ARDS) represent a spectrum of pulmonary alterations resulting from diverse pulmonary insults [1]. ARDS is characterized by acute hypoxemic respiratory failure, arising from pulmonary inflammation rather than cardiogenic pulmonary edema [2]. ARDS is not a transient condition but rather a syndrome that may take days or even weeks to resolve [3], typically leading to high mortality rates. Approximately 10% of ICU admissions and 23% of ventilated patients could be attributed to ARDS [4], and severe cases exhibited mortality rates as high as 45% [5]. Early identification and timely management of ARDS severity are critical to improve clinical outcomes. At present, commonly used laboratory parameters, such as the oxygenation index, are employed to assess the severity and progression of ARDS. However, there is a lack of clinically valuable molecular biomarkers for predicting the severity and mortality of ARDS [1]. Identifying patients who are at risk of deterioration is crucial to optimize the allocation of medical resources and to administer timely supportive treatment. Researchers are actively seeking improved biomarkers for promptly predicting and assessing ARDS patients’ prognosis. Despite significant advances in understanding the pathophysiology of ARDS, identifying reliable predictive molecular biomarkers and developing effective molecular‐based therapies remain a remarkable challenge [6]. Consequently, it is essential to explore personalized biomarkers that can predict ARDS patients’ prognosis and their clinical outcomes.

During the initiation and progression of acute lung injury (ALI), both inflammatory activation and disruption of calcium homeostasis are regarded as pivotal events [7, 8]. Extensive evidence indicates that intracellular calcium (Ca^2+^) acts as a vital messenger, orchestrating inflammatory signaling, apoptosis, and the maintenance of epithelial and endothelial barrier integrity [9–11]. Stromal interaction molecule 1 (STIM1), a key component of calcium signaling located on the endoplasmic reticulum membrane, functions as a calcium sensor that detects reductions in ER Ca^2+^ stores. Upon depletion, STIM1 translocates and activates the plasma membrane channel Orai1, triggering store‐operated calcium entry (SOCE) [12, 13]. This STIM1‐dependent SOCE mechanism is essential for preserving intracellular calcium balance and participates broadly in physiological regulation as well as pathological processes [14, 15].

STIM1 is a newly identified human gene located on chromosome 11p15.5 [16]. Initially found in the endoplasmic reticulum (ER), STIM1 functions as a sensor for ER calcium storage [17], playing a critical role in the physiological and pathological processes of multiple tissues [18]. Recent research has highlighted the significance of STIM1 in cancer, impacting tumor cell genesis, progression, invasion, and metastasis. Notably, STIM1 has shown to significantly influence the behavior of tumor cells [19]. Additionally, Liu et al. [20] emphasized the function of STIM1 in regulating cardiac energy substrate preference and elucidated the molecular mechanisms through which STIM1 modulates cardiac energy metabolism. Collectively, these investigations highlight the role of STIM1 in the pathophysiology of various diseases.

Kaufmann et al. [21] demonstrated the essential role of STIM1 in the store‐operated calcium entry (SOCE) pathway, which in turn regulates mitochondrial function and oxidative phosphorylation (OXPHOS). This regulatory mechanism plays a critical role in preventing oxidative stress and is vital for the proper functionality of pathogenic Th17 cells. The absence of STIM1 results in reduced IL‐17A production, thereby providing protection against pulmonary inflammation instigated by Th17 cells. Moreover, the deficiency of STIM1 causes a shift in gene expression towards a non‐pathogenic Th17 cell profile. Liu et al. [22] pointed out that STIM1 was overexpressed in the serum of patients with influenza A virus (IAV) and in IAV‐infected pulmonary epithelial cells. Silencing STIM1 was proposed as an effective approach to mitigate IAV‐induced oxidative stress, inflammatory responses, apoptosis, and to protect the viability of BEAS‐2B cells. Song et al. [23] demonstrated that STIM1/Orai1 increases endothelial cell (EC) permeability upon exposure to CS and regulates ventilator‐induced lung edema, potentially through calcium‐sensitive PKC*α*. In ROS‐induced A549 cells, elevated expression levels of TRPC1/C3, STIM1, and Orai1, accompanied by increased Ca^2+^ entry via SOCE, resulted in increased expression levels of oxidative, fibrotic, inflammatory, and apoptotic genes, primarily through the extracellular signal‐regulated kinase (ERK) pathway [24]. Higher vascular permeability and enhanced alveolar fluid accumulation are characteristic pathological manifestations of ARDS [25]. STIM1/Orai1 mediates Ca^2+^ influx, which activates PKC*α* and modulates endothelial permeability, inducing pulmonary edema in ventilator‐induced lung injury [23]. These studies demonstrate that STIM1 is critical for the initiation and progression of inflammation and injury in the pulmonary system.

Thus, it is essential to further explore the relationship between ARDS and STIM1. In our study, STIM1 expression level in peripheral blood and bronchoalveolar lavage fluid (BALF) from newly diagnosed ARDS patients was investigated. The GSE database was then utilized to demonstrate STIM1 expression level in ARDS and explore its coexpressed genes and related pathways.

## 2. Materials and Methods

### 2.1. The Overall Framework of This Study Is Presented as a Flow Diagram (Figure 1)

**Figure 1:**
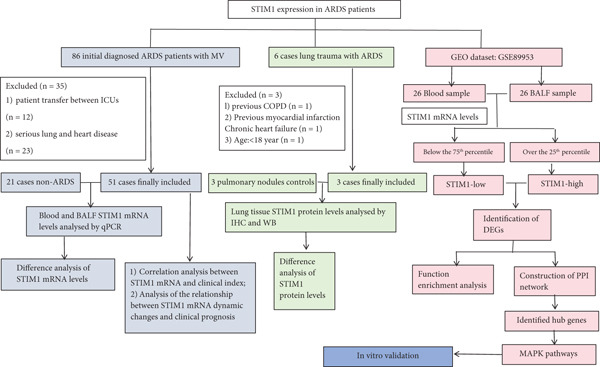
Overall workflow for analyzing STIM1 expression level in ARDS patients.

### 2.2. Patients and Controls

Inclusion criteria: (1) ARDS diagnosis according to the Berlin Definition, including acute onset, PaO₂/FiO₂ ≤ 300 mmHg, positive end‐expiratory pressure (PEEP) ≥ 5 cm H₂O on the first day of ICU admission, bilateral infiltrates on chest radiograph, and absence of heart failure [26]; (2) adults who aged > 18 years; (3) admission to the ICU and initiation of invasive mechanical ventilation on the same day due to ARDS, with mechanical ventilation lasting more than 72 h.

Exclusion criteria: Patients were excluded if they met any of the following conditions: (1) transfer between ICUs; (2) severe lung diseases, such as lung cancer or chronic respiratory conditions (e.g., pulmonary fibrosis or chronic obstructive pulmonary disease); (3) presence of coronary heart disease, atrial fibrillation, heart failure, chronic intestinal disease, autoimmune disease, AIDS, or recent use of immunosuppressant therapy (within 2 weeks).

### 2.3. Study Population and Parameters

This prospective cohort study concentrated on adult patients with ARDS who were admitted to the ICU of Wuhan Third Hospital (Wuhan, China) between May 20, 2023, and March 30, 2024. Patients were monitored from admission until either discharge or death, without any intervention in their medical treatment. A total of 72 patients who were admitted to the ICU of Wuhan Third Hospital required immediate intubation upon admission. Among them, 51 patients met the Berlin clinical criteria for ARDS and were included in the study. Inclusion criteria included hospital admission due to ARDS symptoms resulting from various causes, comprising pneumonia, aspiration, trauma, and sepsis. Survival outcomes were tracked during the ICU stay, with monitoring extending up to 28 days, discharge, or death. Additionally, 21 critically ill patients without ARDS were selected as a control group. These non‐ARDS patients were admitted to the ICU for mechanical ventilation for the following reasons: nine for postoperative monitoring and 12 for severe cerebral infarction and cerebral hemorrhage.

This study also included three ARDS patients with traumatic lung contusion and three with pulmonary nodules to examine pathological changes, immunohistochemical expression, and Western blot protein expression in lung tissue. The three patients with ARDS due to traumatic lung contusion met the Berlin criteria for ARDS and were classified into the ARDS group, and lung tissue samples were obtained postoperatively. The three patients with early‐stage pulmonary nodules were designated as the control group, and tissue samples were extracted from areas distant from the nodule center. All lung tissue samples underwent injury grading, immunohistochemistry (IHC), and Western blot analysis.

The experimental protocol received approval from the Medical Clinical Research Ethics Committee of Wuhan Third Hospital (Approval No. Wu San Yi Lun KY2023‐062), and all participants provided informed consent in compliance with the Declaration of Helsinki.

### 2.4. Clinical Details and Sample Collection

Demographic, clinical, and laboratory data of ARDS patients were extracted from electronic medical records using standardized forms by trained research personnel. Laboratory results obtained within 24 h of admission were analyzed, encompassing gender, age, peripheral white blood cell count, neutrophil count, lymphocyte count, lymphocyte to neutrophil ratio, hemoglobin, platelet count, C‐reactive protein, procalcitonin, serum total Ca^2+^, serum free Ca^2+^, arterial blood pH value, PaO_2_, PaCO_2_, OI, APACHE II score, SOFA scores, mechanical ventilation duration, ICU stay, and 28‐day survival status. Blood and bronchoalveolar fluid samples were collected from 51 ARDS patients and 21 non‐ARDS patients at Wuhan Third Hospital for STIM1 mRNA testing. Local anesthesia with 2% lidocaine was administered for BALF preparation, followed by instillation of 100 mL fractions of room temperature sterile saline into the right middle lobe or left lingular segment of the lung. BALF was aspirated gently using a syringe and transferred to sterile containers after obtaining informed consent from patients. To investigate the association between STIM1 mRNA dynamics and the prognosis of ARDS patients, blood and BALF samples were collected daily for 3 days from 13 ARDS patients, comprising 11 survivors and 2 non‐survivors. Subsequent analysis focused on the dynamic alterations of STIM1 mRNA.

To elucidate the status of lung tissue injury in ARDS and the presence of STIM1 protein, lung tissue samples were obtained from three ARDS patients with lung contusion who underwent lung lobectomy. Additionally, lung tissue samples were obtained from three patients who underwent lung lobectomy for lung nodules, serving as the control group, with the nodule tissue sampled from the periphery of the nodule, specifically from areas distant from the nodule center that appeared relatively normal, avoiding necrotic or fibrotic regions. HE staining was conducted on the collected tissue to assess lung tissue injury. IHC and Western blot analysis were employed to evaluate protein expression levels.

### 2.5. Quantitative RT‐PCR Analysis of STIM1 Expression

Using the Total RNA kit from Wuhan Service Technology Co., Ltd. (China), RNA was extracted from human blood samples. After reverse transcription, RT‐qPCR was performed on the Real‐Time PCR system (Stratagene Mx 3000P) from Agilent Technologies (California, United States). Using the 2^−*ΔΔ*Ct^ method, relative gene expression levels were calculated and all data were normalized to the expression level of β‐actin gene. The primers used in this study are listed in Table 1.

**Table 1:** The primer sequence of STIM1 and β‐actin.

**Gene**	**Forward (5** ^′^ **-3** ^′^ **)**	**Reverse (5** ^′^ **-3** ^′^ **)**
β‐Actin	GGGTATCTCTGCGGCGAATGC	TGGTGGTGGTGGTGGTAG
STIM1	CCACGAAACTACCTTCAACTCCATC	AGTGATCTCCTTCTGCATCCTGTC

### 2.6. Hematoxylin–Eosin Staining

The lung tissue was rinsed with phosphate‐buffered saline (PBS) to eliminate blood stains. Subsequently, it was immersed in a 10% formaldehyde solution for 48 h for fixation. Following fixation, the tissue underwent dehydration, transparency rendering, paraffin embedding, sectioning (4 *μ*m thickness), and HE staining. The morphology of lung tissue was examined using a light microscope, and a pathological assessment for lung injury was conducted.

### 2.7. IHC

IHC was employed to detect the STIM1 protein in lung tissue. Paraffin‐embedded lung tissue was subjected to immunohistochemical analysis using the 11565‐1‐AP STIM1 antibody (Affinity, San Francisco, California, United States). The lung tissue was fixed with 4% paraformaldehyde, embedded into paraffin, and serially sectioned. Specimen staining was performed using the SABC (streptavidin–biotin complex) IHC method. A rabbit anti‐STIM1 polyclonal antibody served as the primary antibody, while a goat anti‐rabbit immunoglobulin was used as the secondary antibody. The enumeration of STIM1‐positive cells was conducted under a light microscope. STIM1 immunoreactive‐positive cells in the sections exhibited a brownish‐yellow color, weakly positive cells appeared yellow, and cells with the same color as the background were regarded negative. Subsequently, an IHC score analysis was carried out utilizing ImageJ software.

### 2.8. Western Blotting

After extracted by lysing 20 mg of lung tissues with radioimmunoprecipitation assay (RIPA) lysis buffer (CST, Danvers, Massachusetts, United States), protein was quantified using the bicinchoninic acid (BCA) protein assay kit (Thermo Fisher Scientific, Waltham, Massachusetts, United States). The protein was then resolved using sodium dodecyl sulfate‐polyacrylamide gel electrophoresis (SDS‐PAGE), followed by polyvinylidene difluoride (PVDF) membrane transfer and membrane blocking with milk and incubation with an anti‐STIM1 antibody (11565‐1‐AP, Affinity) overnight at 4 °C. Anti‐mouse (IgG HampL, Abcam, Cambridge, United Kingdom) or horseradish peroxidase‐conjugated anti‐rabbit (IgG HampL) secondary antibodies were incubated at 37°C for 1 h. Using an enhanced chemiluminescence (ECL) kit (Thermo Fisher Scientific), protein expression was determined and Western blot analysis was performed to quantify protein expression using ImageJ software. All uncropped and original Western blot images used in this study are provided in Figure S1.

### 2.9. Histopathological Scoring of Lung Injury

Lung tissue specimens were independently evaluated by two pathologists who were blinded to the clinical data. A modified scoring system [27, 28] was applied to assess the overall severity of characteristic pathological changes associated with (including hyaline membrane formation, alveolar epithelial injury, inflammatory cell infiltration, interstitial fibrosis, and intra‐alveolar organization). Each specimen was assigned a composite score ranging from 0 to 5 (0: no injury; 5: most severe injury). The total score was subsequently used for statistical analysis.

### 2.10. Dataset Selection and GEO Screening

To ensure transparency and reproducibility, a comprehensive search of the Gene Expression Omnibus (GEO) database was conducted using the keywords “acute respiratory distress syndrome,” “BALF,” and “whole blood.” Several candidate datasets were initially identified (e.g., GSE157789, GSE282919, GSE217763). Among these, GSE89953 was selected for downstream analysis based on the following criteria: (1) inclusion of a well‐defined ARDS cohort with matched controls; (2) sufficient sample size to support robust statistical inference; and (3) availability of mRNA expression profiles from human alveolar macrophages and peripheral blood mononuclear cells (PBMCs), which aligns with our study’s objective of evaluating STIM1 expression in both local (pulmonary) and systemic compartments. Although this dataset used a microarray platform, its biological context was highly consistent with our qPCR‐based experimental framework. Other datasets were excluded due to limited clinical annotation, small sample sizes, lack of relevance to the target population, or inconsistency between the molecular types analyzed and the aims of this study.

### 2.11. Macrophage Culture, LPS Stimulation, and Protein Extraction

The murine macrophage cell line RAW264.7 was kindly provided by the Immunology Research Group of Prof. Fengling Luo, School of Basic Medical Sciences, Wuhan University. Cells were cultured in Dulbecco’s Modified Eagle’s Medium (DMEM; Gibco, United States) supplemented with 10% fetal bovine serum (FBS; Gibco, United States) and 1% penicillin‐streptomycin at 37 °C in a humidified atmosphere containing 5% CO₂. When cells reached approximately 70–80% confluence, they were stimulated with lipopolysaccharide (LPS) (Sigma‐Aldrich, United States) at a final concentration of 1000 ng/mL for 24 h to induce an inflammatory response. Cells cultured under identical conditions without LPS treatment served as the control group. After treatment, cells were washed twice with cold PBS and lysed using RIPA buffer (Beyotime, China) containing protease and phosphatase inhibitors. The lysates were incubated on ice for 30 min and then centrifuged at 12,000×*g* for 15 min at 4 °C. The supernatant was collected, and total protein concentration was determined using the BCA protein assay kit (Thermo Fisher Scientific, United States). The protein samples were stored at −80 °C until further analysis, such as Western blotting.

### 2.12. Statistical Analysis

Continuous variables are presented as mean ± standard error of the mean (SEM) or median (interquartile range [IQR]), as appropriate, and categorical variables are presented as (%). Between‐group comparisons were performed using the chi‐square test, two‐tailed Student’s test, or Mann–Whitney test, as appropriate. Correlations between STIM1 expression levels and clinical severity variables, including the SOFA and APACHE Ⅱ scores, were assessed using Spearman correlation analysis. Receiver operating characteristic (ROC) curve analysis was performed to evaluate the predictive value of STIM1 mRNA levels for 28‐day mortality in patients with ARDS, and the area under the curve (AUC) with the corresponding 95% confidence interval (95% CI) was calculated. The optimal cutoff values were determined, and the corresponding sensitivity and specificity were reported. Kaplan–Meier survival curves were constructed for 28‐day survival according to STIM1 mRNA levels. Binary logistic regression analysis was used to identify independent predictors of 28‐day mortality. Nomogram performance was evaluated by discrimination using the concordance index, calibration curves, and the Hosmer–Lemeshow goodness‐of‐fit test, with 1000 bootstrap resamples. A two‐sided p< 0.05 was considered statistically significant. Figures were generated using R Version 3.6.3 and GraphPad Prism 7.0, and statistical analyses were performed using SAS 9.4. IHC and Western blot quantification were analyzed using ImageJ software.

## 3. Results

### 3.1. Clinical Validation of STIM1 Expression in ARDS Patients

#### 3.1.1. Patient Clinical Features

The characteristics of 51 patients with ARDS and 21 non‐ARDS cases are displayed in Table 2. The ARDS patients had significantly higher blood and BALF STIM1 mRNA levels, as well as higher PCO_2_ and FIO_2_ levels compared with non‐ARDS cases. Additionally, patients with ARDS had lower pH, PO_2_, and P/F (PaO_2_/FiO_2_) levels compared with non‐ARDS cases (*p* < 0.05).

**Table 2:** Characteristics of ARDS patients and non‐ARDS patients.

**Characteristics**	**ARDS patients (** **n** = 51**)**	**Non-ARDS patients (** **n** = 21**)**
Male, sex	37	14
Age, years	71.65 ± 2.21	71.76 ± 2.87
STIM1 mRNA (blood), relative expression	0.36 ± 0.12	0.03 ± 0.01 ^∗^
STIM1 mRNA (BALF), relative expression	0.87 ± 0.14	0.33 ± 0.10 ^∗^
White blood cell count, ×10^9^/L	12.70 ± 0.92	12.76 ± 1.18
Neutrophil count, ×10^9^/L	10.77 ± 0.86	11.00 ± 1.09
Lymphocyte count, ×10^9^/L	1.21 ± 0.15	1.01 ± 0.13
Lymphocyte to neutrophil ratio	0.21 ± 0.07	0.10 ± 0.01
Hemoglobin, g/L	109.43 ± 3.93	101.33 ± 4.73
Platelet count, ×10^9^/L	208.22 ± 18.63	177.95 ± 17.60
C‐reactive protein, mg/L	103.26 ± 12.21	87.76 ± 14.17
Procalcitonin, ng/mL	7.48 ± 2.64	5.14 ± 3.44
Serum total Ca^2+^, mmol/L	2.04 ± 0.03	2.06 ± 0.06
Serum free Ca^2+^, mmol/L	1.19 ± 0.02	1.21 ± 0.03
pH	7.35 ± 0.01	7.41 ± 0.02 ^∗^
PaO_2_, mmHg	90.20 ± 3.56	160.52 ± 7.57 ^∗∗^
PaCO_2_, mmHg	45.89 ± 2.36	38.01 ± 3.09 ^∗^
FiO_2_, %	52.49 ± 2.31	41.67 ± 1.26 ^∗∗^
P/F, mmHg	180.73 ± 7.61	384.82 ± 13.17 ^∗∗^
APACHE II score	28.16 ± 0.94	27.57 ± 0.90
SOFA score	10.14 ± 0.42	9.43 ± 0.77
Mechanical ventilation stay, days	11.37 ± 1.22	14.71 ± 5.17
ICU stay, days	14.57 ± 1.49	17.29 ± 5.07

*Note:* Data were shown as the mean ± SEM.

Abbreviations: NA, not applicable. P/F, PaO_2_/FiO_2_.

^∗^ *p* < 0.05,  ^∗∗^ *p* < 0.01,  ^∗∗∗^ *p* < 0.001 when compared with ARDS patients by two‐tailed Student’s *t* test.

#### 3.1.2. HE, WB, and IHC Analysis of Lung Tissue, qPCR Analysis of Blood and BALF From Patients

HE staining was performed on lung tissue samples from both ARDS and non‐ARDS patients (Figure 2a), and the corresponding lung injury score (Figure 2b). IHC (Figure 2c) indicated that compared with the control group (*n* = 3), the staining signal for STIM1 protein was stronger in the cytoplasm of certain cells in the ARDS group (*n* = 3). Additionally, increased STIM1 protein expression level (Figure 2d) was detected in the ARDS group compared with the control group. The expression level of the STIM1 protein was examined using Western blotting. Increased STIM1 protein expression level (Figure 2e) and higher Western blotting scores (Figure 2f) were found in lung tissue from ARDS patients compared with non‐ARDS cases. Upon admission to the ICU, concurrent with the initiation of mechanical ventilation via intubation, blood and BALF samples were collected from 51 ARDS patients and 21 non‐ARDS ICU patients. STIM1 mRNA level was quantified by RT‐qPCR in both blood and BALF samples. The analysis revealed significantly elevated STIM1 mRNA levels in the blood of ARDS patients compared with non‐ARDS patients (Figure 2g). Similarly, elevated levels of STIM1 mRNA were observed in the BALF of ARDS patients, demonstrating a significant increase compared to non‐ARDS patients in the ICU setting (Figure 2h). Correlation analysis between STIM1 expression (IHC and Western blot) levels and lung injury score. STIM1 protein expression in lung tissues positively correlated with the histological injury score (Figure 2i). The data suggest that STIM1 upregulation is closely associated with the extent of lung injury in ARDS patients.

Figure 2STIM1 was significantly upregulated in the lungs of ARDS patients. (a–d) Lung tissue H&E staining and STIM1 immunohistochemistry (scale bar = 50 *μ*m). (e, f) Western blotting and statistical analysis of STIM1 expression in tissues from human non‐ARDS patients and ARDS patients (*n* = 3 per group). (g, h) Blood and BALF STIM1 mRNA levels were found to be elevated in patients with ARDS. (i) Correlation between STIM1 expression and lung injury score in ARDS. Group comparisons were performed using the Mann–Whitney U test; correlations were assessed using Spearman correlation analysis. *p*:  ^∗^ *p* < 0.05,  ^∗∗^ *p* < 0.01,  ^∗∗∗^ *p* < 0.001.

**Figure:**
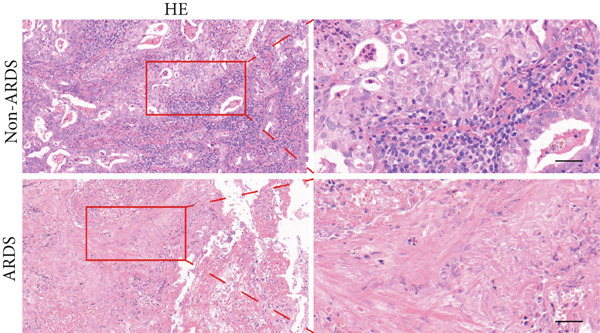
(a)

**Figure:**
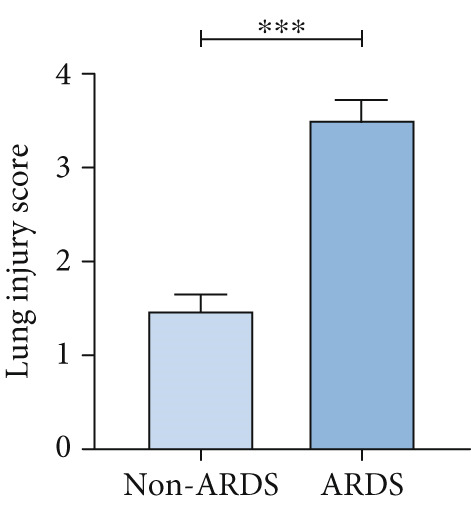
(b)

**Figure:**
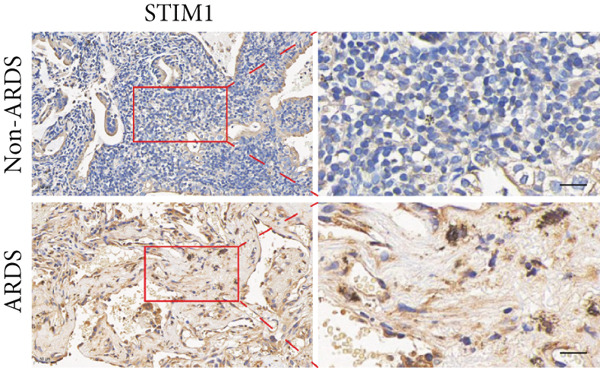
(c)

**Figure:**
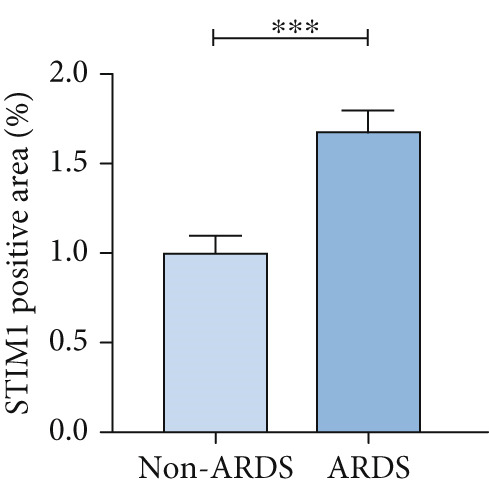
(d)

**Figure:**
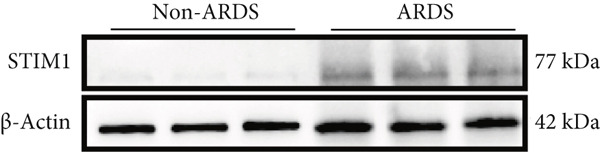
(e)

**Figure:**
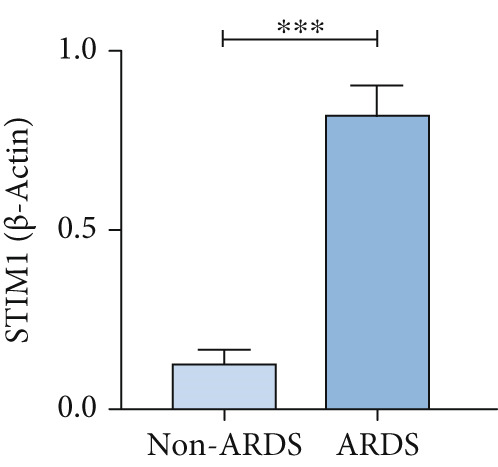
(f)

**Figure:**
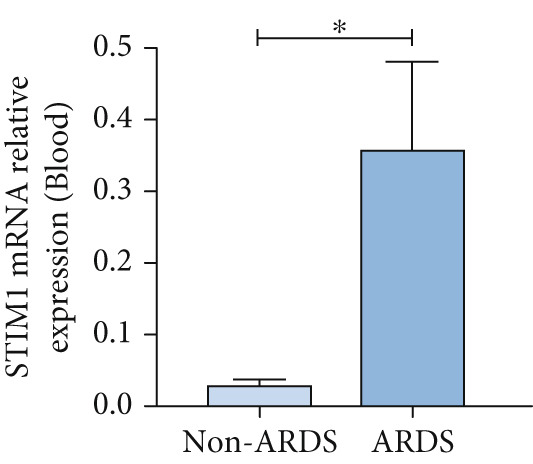
(g)

**Figure:**
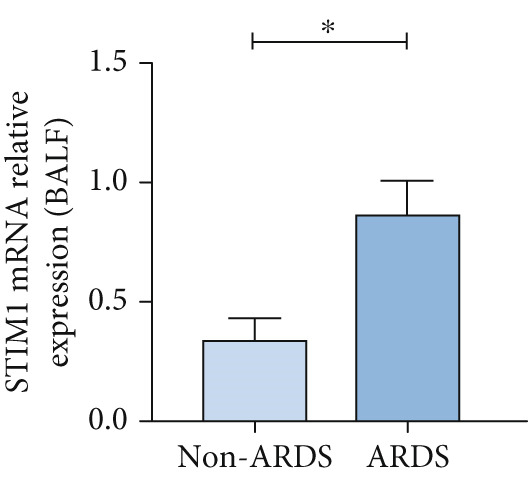
(h)

**Figure:**
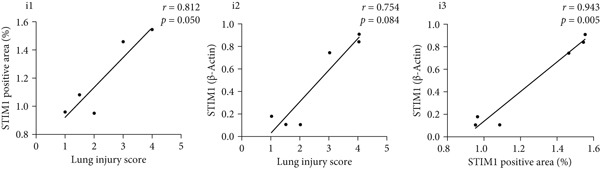
(i)

#### 3.1.3. Expression of STIM1mRNA in Whole Blood of ARDS Patients

Patients with ARDS were categorized into mild, moderate, groups based on their oxygenation index. The findings revealed a positive correlation between the severity of ARDS and STIM1 mRNA level (Figure 3a). A significant difference in STIM1 mRNA level was noted between ARDS patients who survived and those who did not (Figure 3b). Blood samples were collected from 13 patients within the first 3 days of admission, including 11 survived for 28 days, while 2 patients succumbed to the condition. The study tracked STIM1 mRNA level in all ARDS patients (Figure 3c). In the survivors, STIM1 level exhibited a consistent decline over the first 3 days (Figure 3d). Conversely, non‐survivors exhibited a decline in STIM1 mRNA level on the second day, followed by an increase on the third day (Figure 3e).

Figure 3STIM1 mRNA concentrations were measured by qPCR in blood samples in ARDS. (a) In the ARDS group, STIM1 mRNA concentrations varied among the mild group and moderate group. (b) Comparison of blood STIM1 mRNA levels between non‐survivors and survivors in ARDS patients. (c) Examination of blood STIM1 mRNA levels in ARDS patients over the initial 3 days. (d) Evaluation of blood STIM1 mRNA levels in ARDS survivors within the initial 3 days. (e) Assessment of blood STIM1 mRNA levels in non‐survivors of ARDS within the initial 3 days.

**Figure:**
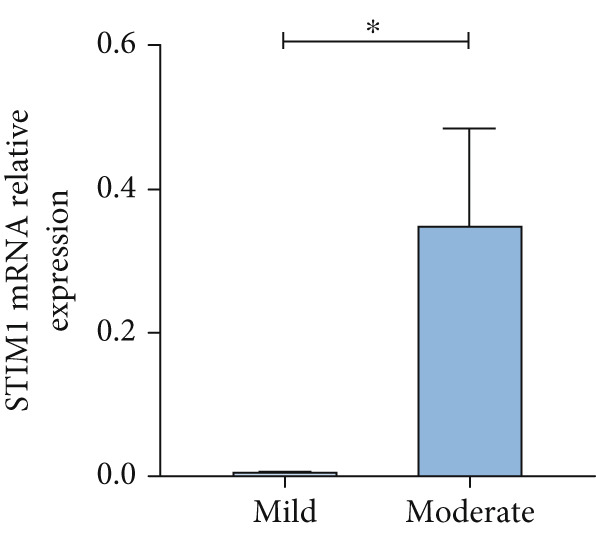
(a)

**Figure:**
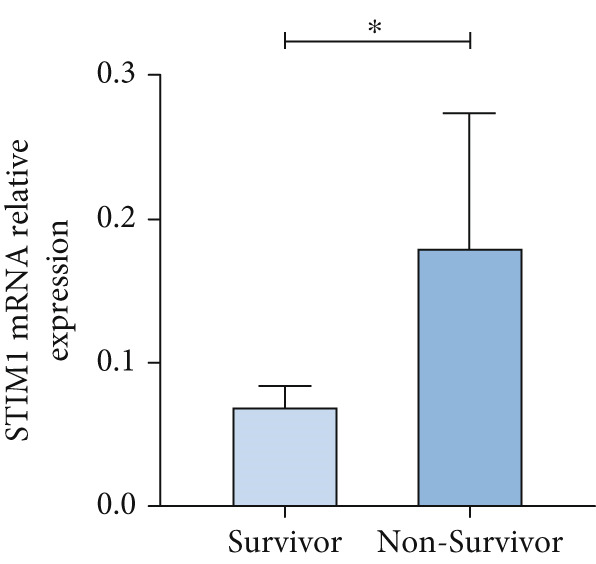
(b)

**Figure:**
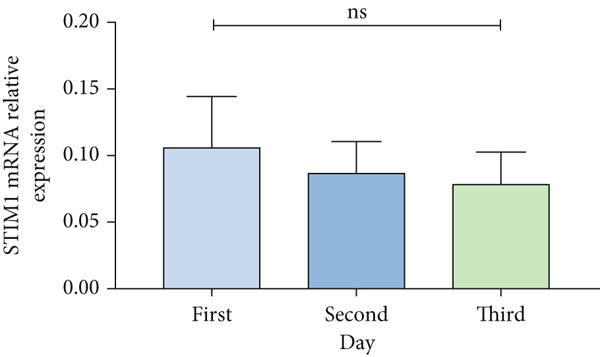
(c)

**Figure:**
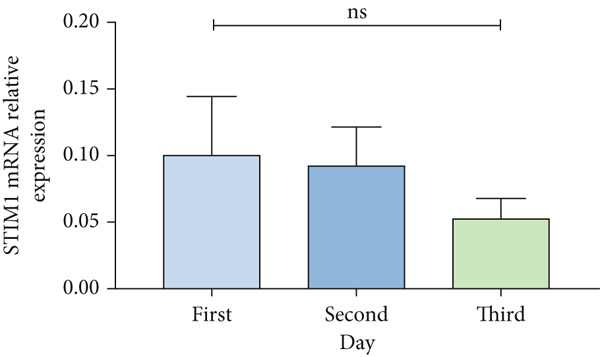
(d)

**Figure:**
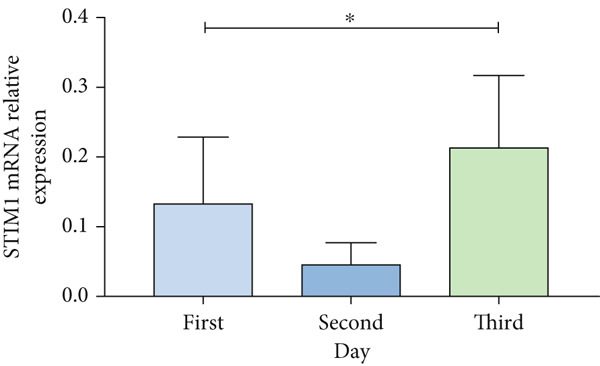
(e)

#### 3.1.4. Expression of STIM1 mRNA in BALF of ARDS Patients

The STIM1 mRNA level in ARDS patients increased in association with the severity of ARDS (Figure 4a). A significant difference in STIM1 mRNA level was identified between surviving and non‐surviving ARDS patients (Figure 4b). Overall, STIM1 mRNA level in BALF decreased within the first 3 days of hospitalization (Figure 4c). In ARDS survivors, STIM1 mRNA level continued to decrease progressively (Figure 4d). In contrast to blood samples, STIM1 mRNA level in BALF exhibited a consistent downward trend in the deceased group (Figure 4e).

Figure 4STIM1 mRNA concentrations were measured by qPCR in BALF samples in ARDS. (a) In ARDS patients, BALF STIM1 mRNA concentrations were assessed in two severity groups: mild and moderate. (b) The levels of STIM1 mRNA in BALF were compared between survivors and non‐survivors of ARDS. (c) The BALF STIM1 mRNA levels were monitored during the initial 3 days of ARDS onset. (d) The analysis of BALF STIM1 mRNA levels in survivors was conducted within the first 3 days of ARDS. (e) BALF STIM1 mRNA levels non‐survivors were also evaluated during the same period.

**Figure:**
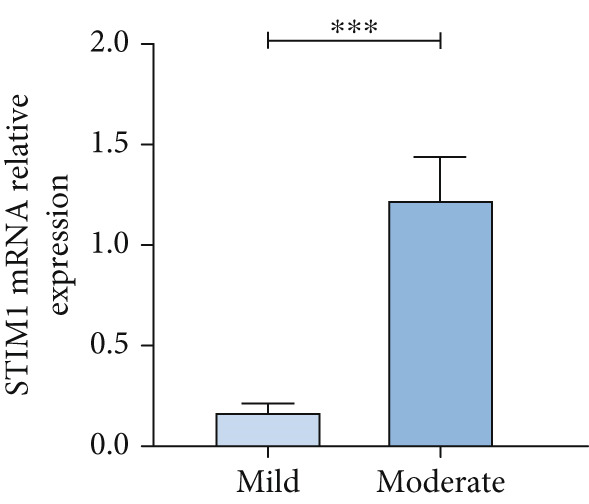
(a)

**Figure:**
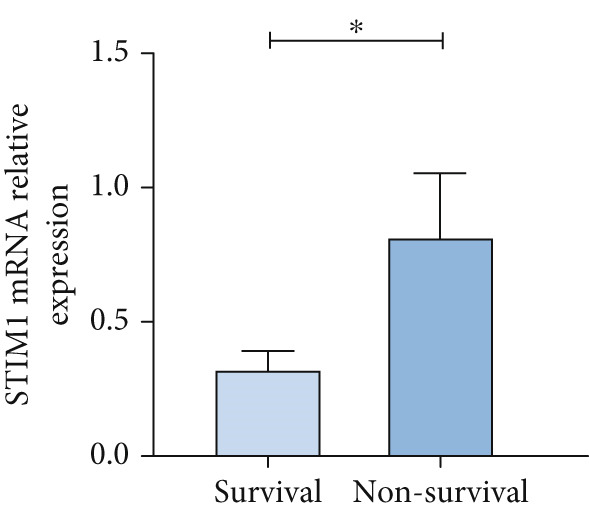
(b)

**Figure:**
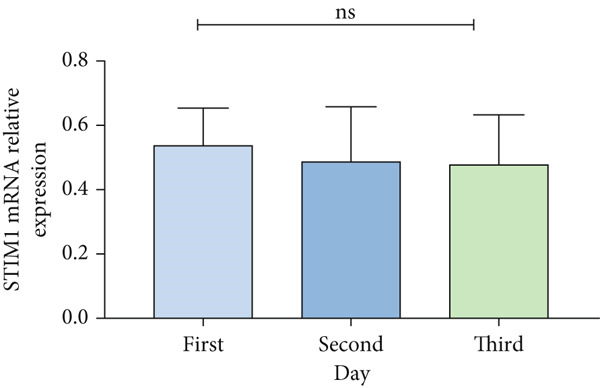
(c)

**Figure:**
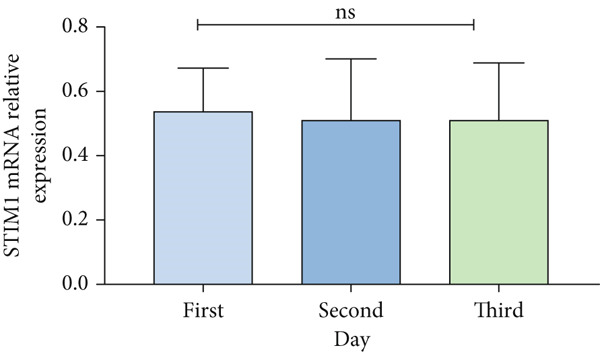
(d)

**Figure:**
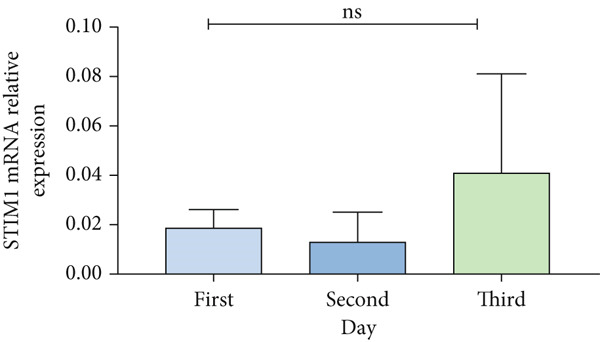
(e)

#### 3.1.5. Correlation Between STIM1 and Clinical Severity Scores in ARDS Patients

Spearman correlation analysis revealed significant positive correlations between STIM1 blood and established clinical severity scores. Specifically, STIM1 blood was moderately correlated with both the SOFA score (*r* = 0.310, *p* = 0.027) (Figure 5a) and APACHE II score (*r* = 0.300, *p* = 0.033) (Figure 5b). In contrast, STIM1 BALF showed no significant correlation with either score (SOFA: *r* = 0.053, *p* = 0.712; APACHE II: *r* = 0.239, *p* = 0.091) (Figure 5c,d).

Figure 5Correlation between blood and BALF STIM1 and APACHE II and SOFA scores. (a) Scatterplot illustrating the correlation between blood STIM1 mRNA concentration and SOFA score. (b) Scatterplot illustrating the correlation between blood STIM1 mRNA concentration and APACHE II score. (c) Scatterplot illustrating the correlation between BALF STIM1 mRNA concentration and SOFA score. (d) Scatterplot illustrating the correlation between BALF STIM1 mRNA concentration and APACHE II score.

**Figure:**
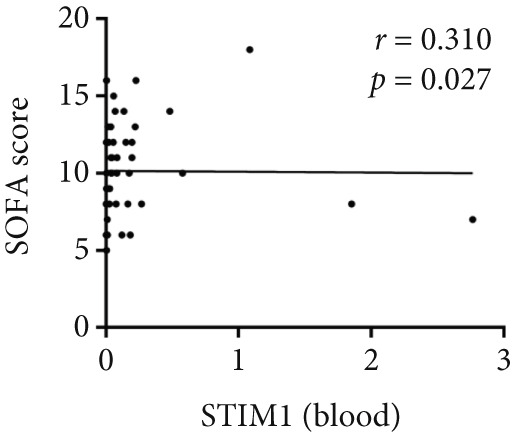
(a)

**Figure:**
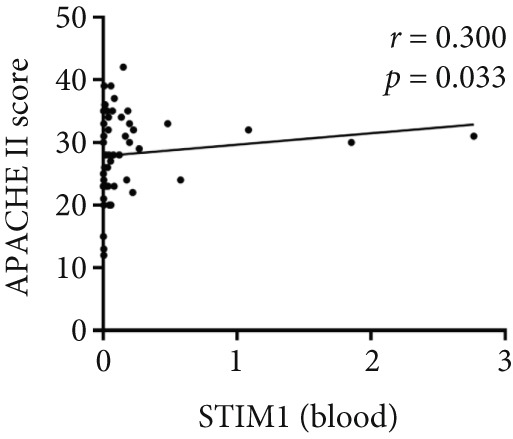
(b)

**Figure:**
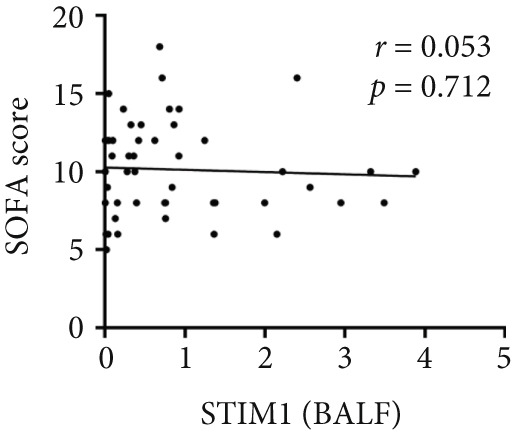
(c)

**Figure:**
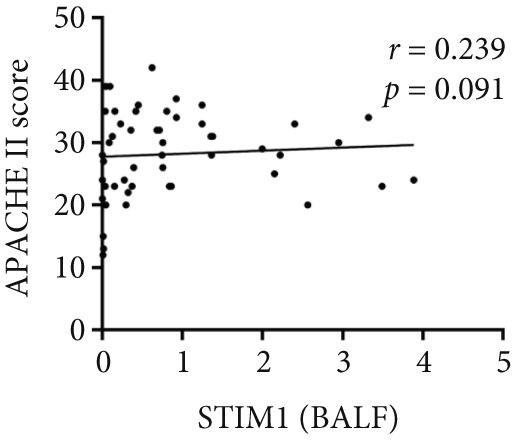
(d)

#### 3.1.6. Patient Characteristics and Univariate Analysis

A total of 51 patients with ARDS were enrolled in this study, among whom 37 (72.5%) experienced the primary outcome of 28‐day mortality or treatment withdrawal, while 14 (27.5%) survived. The baseline characteristics of the study population, stratified by 28‐day survival status, are summarized in Table 3. Univariate analysis revealed that non‐survivors had significantly higher levels of STIM1 mRNA expression both in blood (0.18±0.094 vs. 0.068±0.015,p=0.027); and BALF(0.81±0.25 vs. 0.32±0.070, p=0.0222) compared to survivors. In contrast, no statistically significant differences were observed between survivors and non‐survivors in terms of age, disease severity scores (APACHE II and SOFA), P/F, inflammatory markers (inflammatory index and IL‐6), smoking status, or pathogen type distribution (all *p* > 0.05). These findings suggest that elevated STIM1 expression in both systemic and pulmonary compartments is associated with adverse clinical outcomes in ARDS patients.

**Table 3:** Baseline characteristics of patients stratified by 28‐day survival.

**Characteristics**	**Non-survivors (** **n** = 37**)**	**Survivors (** **n** = 14**)**	***p* value**
Demographics			
Age, years	71.92 ± 2.77	70.93 ± 3.52	0.844
STIM1 expression			
STIM1 mRNA (blood)	0.18 ± 0.094	0.068 ± 0.015	0.027 ^∗^
STIM1 mRNA (BALF)	0.81 ± 0.25	0.32 ± 0.070	0.022 ^∗^
Disease severity scores			
APACHE II score	27.30 ± 1.08	30.43 ± 1.80	0.138
SOFA score	10.16 ± 0.54	10.07 ± 0.59	0.925
P/F	179.48 ± 9.63	184.04 ± 11.44	0.792
Inflammatory markers			
Inflammatory index	‐0.084 ± 0.29	0.22 ± 0.47	0.580
IL‐6, pg/mL	11.18 ± 0.98	9.70 ± 1.81	0.447
Other variables			
Smoking status	0.41 ± 0.11	0.71 ± 0.22	0.164
Pathogen type	1.57 ± 0.24	1.00 ± 0.18	0.068

*Note:* Data presented as mean ± standard error of mean (SEM). The inflammatory index was derived using the formula: Inflammatory index = [WBC + log_10_(CRP) + log_10_(PCT)]. WBC, CRP, and PCT measurements were standardized prior to index calculation.

*p < 0.05, **p < 0.01, and ***p < 0.001.

#### 3.1.7. Multivariable Linear Regression: STIM1 Blood as an Independent Predictor of Disease Severity

Multivariable linear regression analyses were performed to assess the independent relationship between STIM1 expression levels and disease severity, as measured by the APACHE II score (Table 4). Two hierarchical models were constructed to sequentially adjust for potential confounding factors. In Model 1, adjusted for demographic characteristics, clinical variables, and inflammatory status, blood STIM1 mRNA expression demonstrated a significant positive association with APACHE II scores (B = 5.735, SE = 2.114, *p* = 0.010), indicating that each unit increase in blood STIM1 was associated with a 5.74‐point elevation in the APACHE II score. Age also emerged as a significant independent predictor in this model (B = 0.182, SE = 0.062, *p* = 0.006). In contrast, BALF STIM1 expression was not significantly associated with disease severity (B = 0.489, SE = 0.915, *p* = 0.596). In the fully adjusted Model 2, which additionally incorporated the clinical severity grade, blood STIM1 maintained its significant independent association with higher APACHE II scores (B = 5.797, SE = 2.241, *p* = 0.013). The association of age with disease severity remained significant (B = 0.184, SE = 0.068, *p* = 0.010), while BALF STIM1 expression continued to show no significant association (*p* = 0.598). Notably, the clinical severity grade itself was not a significant predictor in the final model (*p* = 0.926). These results robustly indicate that elevated STIM1 expression in the blood, but not in the alveolar lavage fluid, serves as an independent biomarker of disease severity in ARDS patients, providing prognostic information beyond that captured by traditional clinical assessments.

**Table 4:** Multiple linear regression analysis for APACHE II score prediction.

**Predictor**	**Model 1**		**Model 2**	
	**B (SE)**	***p* value**	**B (SE)**	***p* value**
STIM1 mRNA (Blood)	5.735 (2.114)	0.010 ^∗^	5.797 (2.241)	0.013 ^∗^
STIM1 mRNA (BALF)	0.489 (0.915)	0.596	0.492 (0.927)	0.598
Age, years	0.182 (0.062)	0.006 ^∗^	0.184 (0.068)	0.010 ^∗^
Oxygenation index (P/F)	0.034 (0.018)	0.062	0.037 (0.042)	0.374
Smoking status	− 0.521 (1.273)	0.684	− 0.510 (1.295)	0.696
28‐day mortality	4.106 (2.130)	0.061	4.174 (2.277)	0.074
Pathogen type	− 0.855 (0.710)	0.235	− 0.870 (0.737)	0.245
Inflammatory index	0.740 (0.600)	0.224	0.744 (0.608)	0.228
IL‐6, pg/mL	0.041 (0.158)	0.796	0.043 (0.161)	0.793
Clinical severity grade	—	—	0.341 (3.641)	0.926
Constant	7.389 (6.618)	0.271	5.984 (16.428)	0.718

*Note:* Dependent variable: APACHE II score. Data presented as unstandardized coefficients (B) with standard errors (SE). Model 1: adjusted for demographic and clinical variables. Model 2: Model 1 + clinical severity grade.

*p < 0.05, **p < 0.01, and ***p < 0.001.

#### 3.1.8. Multivariable Analysis for 28‐Day Mortality Prediction

Multivariable binary logistic regression analyses were performed to assess the association between STIM1 expression levels and 28‐day mortality (Table 5).

**Table 5:** Binary logistic regression analyses for predictors of 28‐day mortality.

**Predictor**	**Model 1**		**Model 2**	
	**OR (95% CI)**	***p* value**	**OR (95% CI)**	***p* value**
STIM1 mRNA (Blood)	<0.001 (<0.001–17.889)	0.107	<0.001 (<0.001–2.219)	0.063
STIM1 mRNA (BALF)	0.347 (0.097–1.243)	0.104	0.339 (0.078–1.474)	0.149
Age, years	0.981 (0.921–1.045)	0.551	0.946 (0.875–1.023)	0.162
Oxygenation index (P/F)	1.001 (0.981–1.022)	0.926	0.942 (0.893–0.993)	0.028 ^∗^
APACHE II score	1.201 (1.029–1.402)	0.020 ^∗^	1.237 (1.034–1.481)	0.020 ^∗^
SOFA score	0.775 (0.502–1.198)	0.251	0.736 (0.446–1.217)	0.232
Smoking status	1.788 (0.576–5.550)	0.315	1.184 (0.333–4.208)	0.794
Pathogen type	0.564 (0.227–1.400)	0.217	0.628 (0.229–1.723)	0.367
Inflammatory index	1.530 (0.758–3.089)	0.235	1.029 (0.510–2.075)	0.936
IL‐6, pg/mL	0.863 (0.727–1.025)	0.094	0.863 (0.727–1.025)	0.094
Clinical severity grade	—	—	0.003 (0.000‐0.535)	0.028 ^∗^
Constant	2.792	0.735	8.08e9	0.040

*Note:* Dependent variable: 28‐day mortality (death/withdrawal = 1, survival = 0). Data presented as odds ratios (OR). Model 1: adjusted for demographic, clinical, and inflammatory variables. Model 2: Model 1 + clinical severity grade.

*p < 0.05, **p < 0.01, and ***p < 0.001.

In Model 1, adjusted for demographic characteristics, clinical variables, and inflammatory status, neither STIM1 blood expression (OR 0.000, 95% CI 0.000–17.889, *p* = 0.107) nor BALF STIM1 expression (OR 0.347, 95% CI 0.097–1.243, *p* = 0.104) was significantly associated with mortality. In this model, only the APACHE II score demonstrated a significant independent association with increased odds of mortality (OR 1.201, 95% CI 1.029–1.402, *p* = 0.020).

In the fully adjusted Model 2, which further incorporated the clinical severity grade, blood STIM1 expression showed a trend towards significance but did not reach the conventional threshold (OR 0.000, 95% CI 0.000–2.219, *p* = 0.063). BALF STIM1 expression remained non‐significant (OR 0.339, 95% CI 0.078–1.474, *p* = 0.149). Conversely, three established clinical parameters were confirmed as significant independent predictors of mortality in this comprehensive model: the APACHE II score (OR 1.237, 95% CI 1.034–1.481, *p* = 0.020), the oxygenation index (P/F) (OR 0.942, 95% CI 0.893–0.993, *p* = 0.028), and the clinical severity grade (OR 0.003, 95% CI 0.000–0.535, *p* = 0.028).

Elevated blood STIM1 expression serves as a robust biomarker reflecting the severity of illness in ARDS patients, yet it does not independently predict ultimate mortality beyond the information captured by established clinical severity scores.

#### 3.1.9. STIM1 mRNA AUC and 28‐Day Survival Results in Patients With ARDS

A total of 51 patients with ARDS were enrolled in this study, among whom 37 (72.5%) experienced the primary outcome of 28‐day mortality or treatment withdrawal, while 14 (27.5%) survived. The AUC for STIM1 mRNA level in blood in predicting 28‐day mortality was 0.58 (95% CI 0.42–0.74), while the AUC for STIM1 mRNA level in BALF was 0.52 (95% CI 0.35–0.69) (Figure 6a). Cases were categorized into low and high STIM1 mRNA groups based on specific cutoff values for STIM1 mRNA level in blood and BALF (Table 6). The brown dotted line in the survival curves represents ARDS patients with low blood STIM1 mRNA levels (< 0.0133 ng/mL), while the red dotted line represents those with high blood STIM1 mRNA levels (≥ 0.0133 ng/mL) (Figure 6b). Similarly, for BALF STIM1 mRNA levels, the brown dotted line indicates the survival curve for patients with low levels (< 0.3107 ng/mL), and the red dotted line represents the survival curve for those with high levels (≥ 0.3107 ng/mL) (Figure 6c). The risk of mortality for ARDS patients with high blood STIM1 mRNA levels (≥ 0.0133 ng/mL) was 5.94 times higher than that for patients with low blood STIM1 mRNA levels (< 0.0133 ng/mL). Additionally, the mortality risk was 2.36 times higher in the high BALF STIM1 mRNA level group (≥ 0.3107 ng/mL) compared with the low BALF STIM1 mRNA level group (< 0.3107 ng/mL).

Figure 6The STIM1 mRNA AUC and survival curve in ARDS. (a) The study analyzed the AUC for STIM1 mRNA levels in both blood and BALF. The identified cutoff value for predicting 28‐day mortality in ARDS patients was 0.0133 ng/mL for blood STIM1 mRNA and 0.3107 ng/mL for BALF STIM1 mRNA. (b) The survival curve based on blood STIM1 mRNA levels over a 28‐day period indicated statistical significance, *p* < 0.05. (c) The survival curve based on BALF STIM1 mRNA levels over a 28‐day period, *p* < 0.05.

**Figure:**
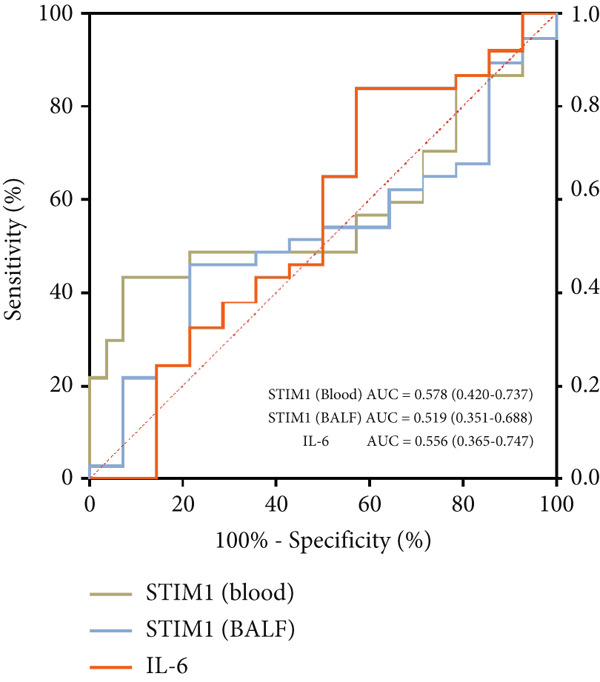
(a)

**Figure:**
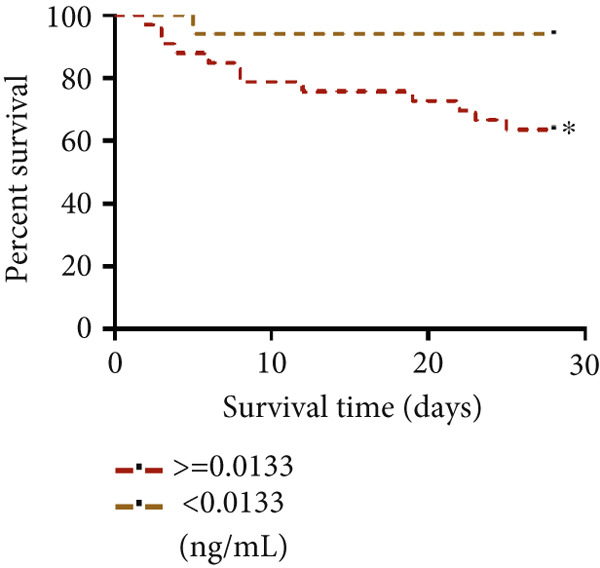
(b)

**Figure:**
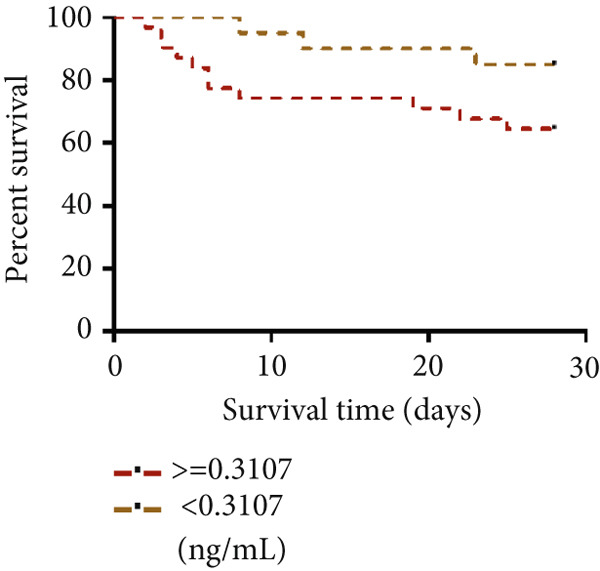
(c)

**Table 6:** The value of predicting 28‐day mortality in patients with ARDS.

**Parameter**	**AUC (95% CI)**	**Cutoff value**	**SE (%)**	**SP (%)**	**PPV (%)**	**NPV (%)**
STIM1 mRNA (Blood)	0.578	0.013	0.929	0.361	0.592	0.836
STIM1 mRNA (BALF)	0.519	0.311	0.786	0.245	0.510	0.534
IL‐6	0.556	15.140	0.429	0.838	0.726	0.595

#### 3.1.10. Nomogram Development and Validation

A nomogram was developed to predict the severity of ARDS. This nomogram highlights the four factors with the highest AUC values from the ROC curve: STIM1 mRNA expression level in blood and BALF, the APACHE II score, and the SOFA score (Figure 7a). The likelihood of a patient developing severe ARDS was estimated by summing the scores for each risk factor. The mean absolute error for predicting ARDS severity using this nomogram was 0.077, indicating a deviation rate in its predictions (Figure 7b). Similarly, a predictive nomogram for assessing mortality risk in severe ARDS patients was developed, incorporating blood STIM1 mRNA level, BALF STIM1 mRNA level, APACHE II score, and SOFA score (Figure 7c). The mean absolute error for predicting mortality risk in ARDS patients using this nomogram was 0.055 (Figure 7d). The nomogram was developed and internally validated using complete cases with full data available for all included predictors (n = 43).

Figure 7Nomogram developed to predict ARDS severity and mortality. (a) Nomogram developed to predict ARDS severity. (b) The deviation rate of the nomogram in predicting the severity of ARDS disease. (c) Nomogram developed to predict ARDS patient mortality risk. (d) The deviation rate of the nomogram in predicting the mortality risk of ARDS disease. APACHE II, Acute Physiology and Chronic Health Evaluation II; SOFA, sequential organ failure assessment. Points: the individual scores corresponding to each predictor in the model in different groups/values. Total points: the total score of each score corresponding to the values of all predictors. Probability: the predicted probability of event occurrence at the corresponding total point value.

**Figure:**
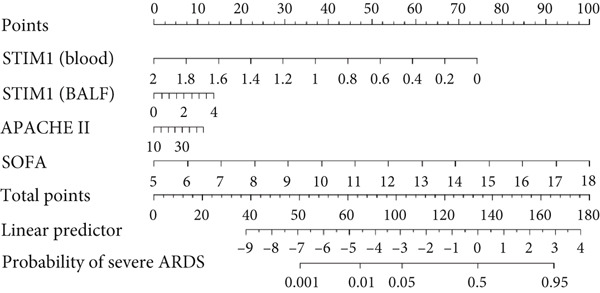
(a)

**Figure:**
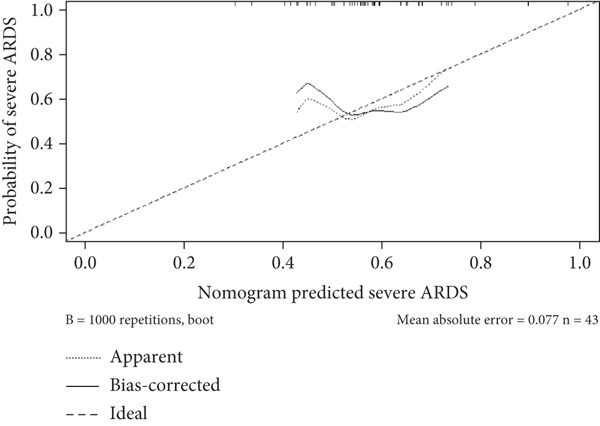
(b)

**Figure:**
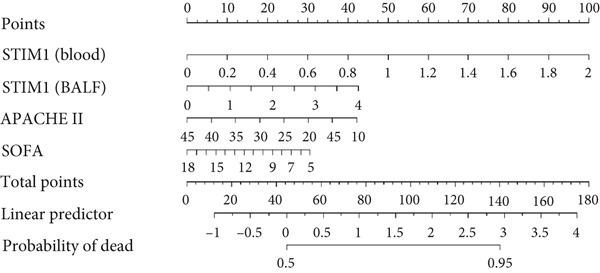
(c)

**Figure:**
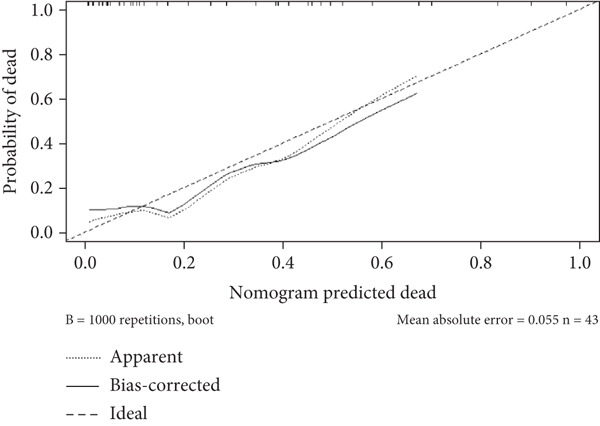
(d)

### 3.2. Bioinformatics‐Based Prediction and Pathway Analysis

#### 3.2.1. Validation of the Correlation Between STIM1 and Hub Genes

The analysis of the STIM1‐related dataset (GSE89953), comprising 26 blood and 26 BALF samples from ARDS patients, identified distinct STIM1 mRNA expression patterns between the two sample types (Figure S2A). Hierarchical clustering delineated clear subgroups of high and low STIM1 expression levels, highlighting significant differential expression levels in the ARDS cohort (Figure S2B). Post‐normalization of microarray data, volcano plot revealed 709 upregulated and 371 downregulated differentially expressed genes (DEGs) (adjusted *p* value < 0.05, |log2 fold change (FC)| > 1) (Figure S2C). A heat map of the top 50 DEGs, based on adjusted *p* value permutations, illustrated distinct expression clusters across samples, confirming the transcriptional heterogeneity linked to STIM1 expression level (Figure S2D).

A coexpression analysis was conducted to identify genes coexpressed with STIM1, and a protein–protein interaction (PPI) network was thereafter constructed using the STRING database. The maximum intersection of this network was retained, and the CytoHubba and MCODE plugins in Cytoscape were used to identify the mitogen‐activated protein kinase (MAPK) pathway and its associated submodules, which were most closely linked to STIM1 (Figure S3). Further analysis of the differentially expressed mRNAs in the STIM1 network revealed that the MAPK pathway was one of the top 25 enriched GO and KEGG pathways (Figure 8a). By integrating KEGG pathway analysis and PPI network screening, the MAPK pathway was selected for further investigation. The regulation of the STIM1 gene in ARDS by the MAPK pathway was initially identified through database screening. Subsequently, this pathway was validated in ARDS and non‐ARDS lung tissues (Figure 8b). Western blot assay was performed to investigate the levels of phospho‐p38, phospho‐ERK, and phospho‐JNK in lung tissues (Figures 8c, 8d, and 8e). The levels of phospho‐ERK and phospho‐JNK were significantly upregulated compared with the control group.

Figure 8Construction of a PPI network of genes that coexpressed with STIM1 and validation of MAPK pathway in ARDS lung tissue. (a) Top 25 enriched Gene Ontology (GO) pathways and KEGG pathways of differentially expressed mRNAs related to the STIM1 network. (b) Quantitative statistical analysis of related protein expression. (c–e) The WB score was higher in ARDS lung tissues than in non‐ARDS lung tissues, using ImageJ analysis.

**Figure:**
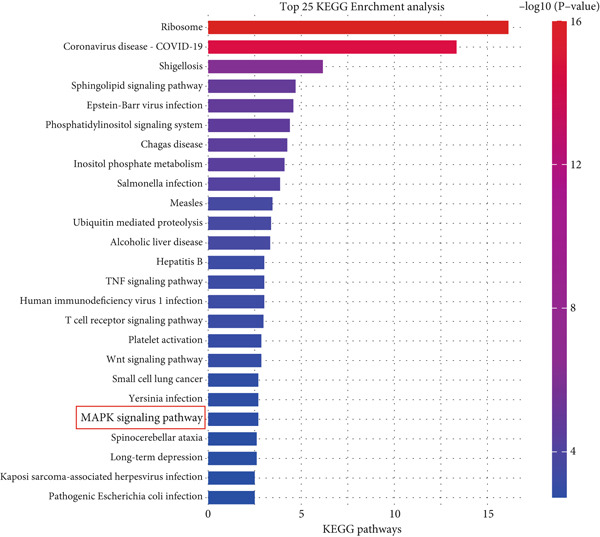
(a)

**Figure:**
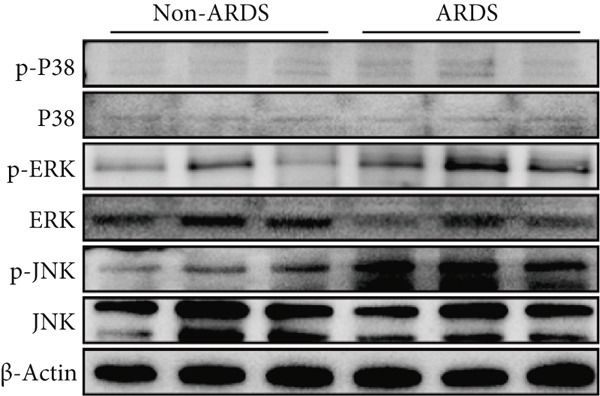
(b)

**Figure:**
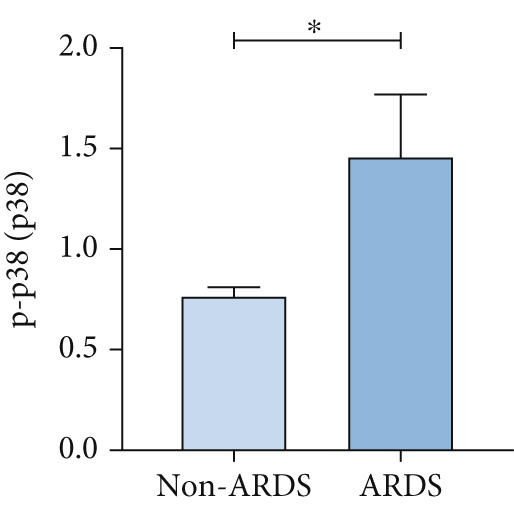
(c)

**Figure:**
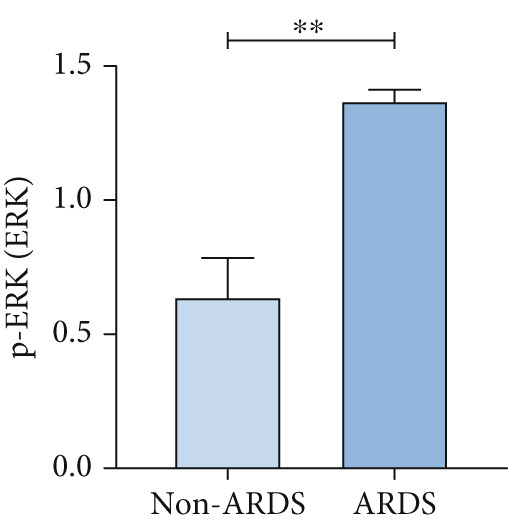
(d)

**Figure:**
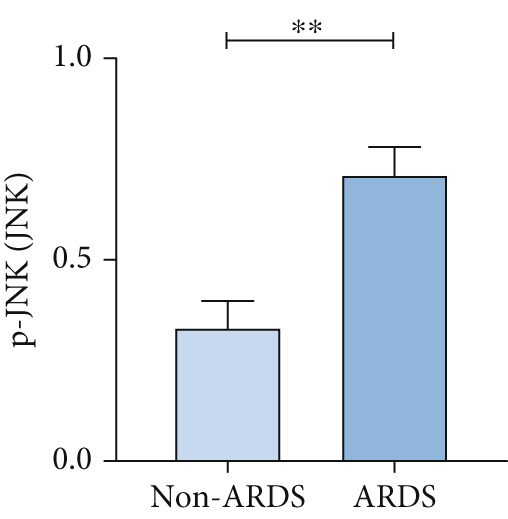
(e)

### 3.3. Mechanistic Confirmation in Cellular Models

#### 3.3.1. Validation of the MAPK Pathway in LPS‐Induced Macrophages

To further validate STIM1 expression level in macrophages and its association with the MAPK pathway, Western blot assay was performed to analyze levels of phospho‐p38, phospho‐ERK, and phospho‐JNK in macrophages (Figures 9a, 9b, 9c, 9d, and 9e). The levels of phospho‐ERK and phospho‐JNK were significantly elevated compared with the control group.

Figure 9Validation of MAPK pathway in macrophages. (a) The expression of phosphorylation of STIM1, p‐JNK, JNK, p‐P38, p38, p‐ERK, ERK in ARDS and non‐ARDS lung tissue. (b–e) Quantitative statistical analysis of related protein expression.

**Figure:**
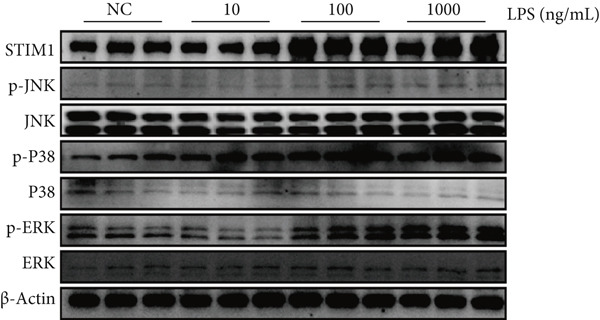
(a)

**Figure:**
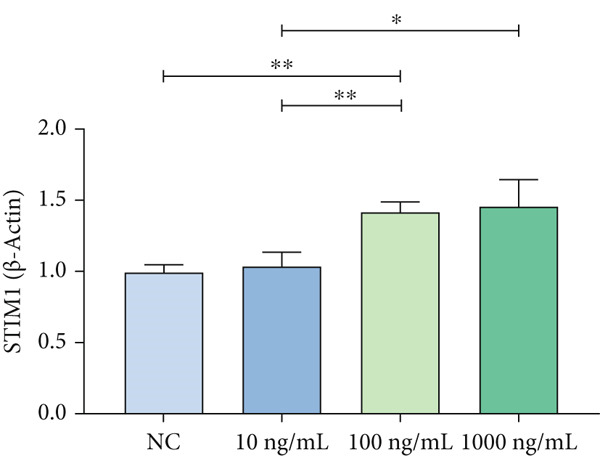
(b)

**Figure:**
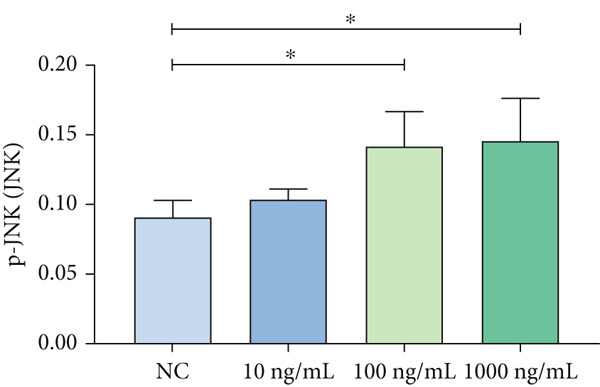
(c)

**Figure:**
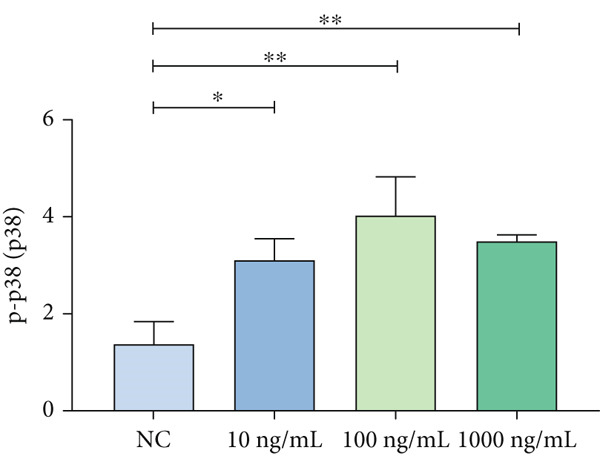
(d)

**Figure:**
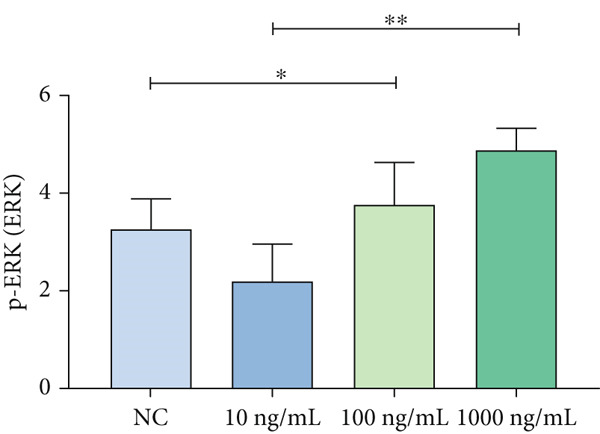
(e)

## 4. Discussion

Elevated STIM1 mRNA expression was detected in both peripheral blood and BALF samples from patients with ARDS. The STIM1 transcript level showed a positive correlation with disease severity and patient mortality. Consistent with these findings, analysis of the GEO dataset further confirmed high STIM1 expression in ARDS and revealed a set of coexpressed genes and enriched signaling pathways, most notably involving MAPK‐related molecular functions.

ARDS is a critical condition that can result from diverse pulmonary and non‐pulmonary causes. The mortality rate for ARDS is notably high, typically ranging from 35% to 46% [4], drawing significant global attention. At present, a dependable biomarker for predicting ARDS patients’ prognosis upon ICU admission is lacking. The present study identified STIM1 mRNA as a potential marker for predicting the severity and mortality risk in ARDS patients. The following evidence supports these findings: Firstly, elevated STIM1 level was detected in the blood, BALF, and lung tissues of ARDS patients compared with non‐ARDS cases. The STIM1 mRNA level was associated with disease severity, as found in both blood and BALF samples. Furthermore, STIM1 mRNA level was correlated with ARDS progression and patient outcomes. Notably, lower STIM1 mRNA levels were identified in surviving ARDS patients compared with non‐survivors. In addition, blood STIM1 mRNA level showed a positive correlation with the SOFA and APACHE II scores, both of which are used in the ICU to evaluate patient severity and mortality risk. Distinct patterns were noted between survival and non‐survival cohorts during the first 3 days. The survival group displayed a downward trend in blood STIM1 mRNA level, whereas the non‐survival group exhibited an upward trend by the third day. The ROC analysis was conducted using STIM1 mRNA. Although the sensitivity and specificity for predicting mortality were suboptimal, survival curves were subsequently analyzed based on the cutoff values from the ROC curve. The results revealed a significant difference in the 28‐day survival rate between patients with high and low levels of STIM1 mRNA. Survival curve analysis indicated that patients with elevated STIM1 mRNA levels in both blood and BALF upon admission had poorer survival outcomes compared with those with lower levels. Predictive models incorporating STIM1 mRNA expression levels were then developed to assess the severity of ARDS and mortality risk, demonstrating strong predictive accuracy.

A comparison of STIM1 levels in blood and BALF samples indicated a higher BALF concentration than in blood, suggesting potential STIM1 accumulation in the lungs of patients with ARDS. Notably, the findings revealed that blood STIM1 level exhibited greater predictive accuracy for disease severity and mortality risk compared with BALF STIM1 level. Particularly in the ARDS mortality group, blood STIM1 mRNA expression showed consistent superiority trend over BALF levels during the initial 3 days, and a significant correlation was identified between blood STIM1 mRNA levels and two established scoring systems, while no such correlation was found in BALF samples. These findings suggest that blood STIM1 level provided a more reliable means of assessing ARDS severity and mortality risk, potentially providing a more accurate reflection of systemic conditions. Several factors may explain this disparity, compartmentalized kinetics between the alveolar and systemic compartments may explain our divergent findings (declining BALF STIM1 vs. persistently rising blood STIM1 in non‐survivors). In ARDS, the host response is well recognized to be compartmentalized, with biomarker levels and prognostic associations differing between BALF and plasma. Multiple studies show that alveolar and systemic inflammatory signals are only partially coupled, and their time courses can diverge [29]. As epithelial and endothelial injury disrupt the alveolar–capillary barrier, lung‐derived mediators more readily spill over into the circulation, where higher plasma levels (e.g., sRAGE, SP‐D) track with severity and mortality [30]. Conversely, airspace concentrations may decline over time due to macrophage‐mediated clearance, proteolysis, mucociliary transport, and dilution from ongoing edema/ventilation, despite persistent systemic inflammation [31]. These mechanisms provide a biologically plausible framework in which blood STIM1 continues to accumulate (reflecting ongoing systemic activation and/or translocation across a leaky barrier), while BALF STIM1 decreases as the alveolar compartment undergoes dynamic clearance or cellular depletion. Although STIM1 is an intracellular ER protein, similar compartmentalized kinetics have been described for other epithelium‐derived markers, supporting our interpretation and highlighting the need for longitudinal, paired sampling across compartments [32]. We also acknowledge that the number of non‐survivor cases with longitudinal sampling was limited, which may influence the interpretation of temporal STIM1 dynamics between compartments. In contrast, blood samples are more easily accessible, less invasive, and less prone to external influences. Therefore, blood STIM1 level should be prioritized as the primary marker for disease assessment, and BALF analysis served as a complementary tool in clinical evaluation.

When comparing the AUC values of STIM1 (in both blood and BALF) and IL‐6 for predicting 28‐day mortality, STIM1 showed a predictive performance similar to that of IL‐6, as indicated by the overlap of their AUC confidence intervals. However, subsequent multivariable analyses revealed a distinct role for STIM1. Unlike IL‐6, STIM1 did not remain an independent predictor of mortality when included in a model containing the APACHE II score. Nevertheless, after adjustment for IL‐6 and other confounding variables, circulating STIM1 remained significantly and independently correlated with disease severity (APACHE II score). These findings suggest that, rather than serving primarily as a mortality predictor, STIM1 may function as a complementary biomarker that more accurately reflects real‐time disease severity.

ARDS is characterized by an acute and diffuse inflammatory lung injury, leading to enhanced alveolar capillary permeability, increased lung weight, and loss of aerated lung tissue [33], resulting in significant hypoxia. Despite the use of various protective strategies, such as extracorporeal membrane oxygenation, prone positioning ventilation, and continuous high‐volume hemofiltration, the mortality rate of ARDS remains remarkable [34]. The pathophysiology of ARDS involves various mechanisms that were partially understood, although several aspects remain elusive. Evidence suggests that inhibiting store‐operated channels (SOCs) through STIM1 inhibition reduces pro‐inflammatory cytokine production in mice exposed to lipopolysaccharides (LPS), thereby preventing lung injury [35]. Research has demonstrated that Paraquat specifically targets the STIM1‐TRPC1 axis, leading to an influx of extracellular calcium in pulmonary epithelial cells. This results in an overload of intracellular Ca^2+^ and ultimately contributes to the development of pulmonary fibrosis [36]. It was previously confirmed that BTP2, an inhibitor of calcium channels, can alleviate lung injury [37]. Previous research has shown that the deletion of STIM1 can be protective against lung inflammation caused by Th17 cells [21].

STIM1 is an ER calcium sensor that plays a crucial role in linking LPS‐induced reactive oxygen species (ROS), calcium oscillations, and EC dysfunction. Calcium ions (Ca^2+^) serve as important second messengers in various cell types, influencing a wide range of cellular processes, including cell survival [21]. It was recently demonstrated that the STIM1 protein plays novel and unexpected physiological and pathophysiological roles in multiple tissues [38]. The clinical phenotype of patients with STIM1 deficiency is characterized by a combination of immunodeficiency, autoimmune disease, congenital myopathy, and ectodermal dysplasia [39]. During ER stress, the upregulation of two components of store‐operated calcium entry (SOCE), STIM1 and STIM2, as well as the SOCE channel Orai3, led to ROS production and contributed to cell death due to oxidative stress [40]. In ECs, Ca^2+^ signaling is essential for maintaining barrier function and regulating vascular tone [41]. LPS‐challenged mice exhibit characteristic pathological signs of ALI, including increased levels of circulating inflammatory cytokines, elevated pulmonary EC activation, enhanced vascular permeability, and increased accumulation of alveolar fluid. The overproduction of ROS in ECs has shown to contribute to LPS‐induced ALI [25]. STIM1 signaling plays a role in the death of pulmonary vascular ECs. Research by Rajesh Kumar Gandhirajan and colleagues using Stim1*Δ*EC mice demonstrated that STIM1‐mediated Ca^2+^ entry could be a crucial factor in the activation of EC and necrotic cell death induced by LPS. These findings contribute to the growing body of evidence indicating that STIM‐mediated Ca^2+^ entry negatively impacts various pathophysiological conditions [35]. In ventilator‐induced lung injury, Ca^2+^ overload in ECs increases endothelial permeability, leading to pulmonary edema [23]. In cases of ALI due to severe sepsis, the disruption of Ca^2+^ homeostasis in ECs is a critical factor, influencing their functionality [42]. Sepsis‐induced ALI is linked to elevated intracellular Ca^2+^ concentrations. This condition is driven by STIM1 and the Ca^2+^‐release‐activated Ca^2+^ channel protein 1 (Orai1), which together facilitate store‐operated Ca^2+^ entry (SOCE) [43]. Debroy et al. demonstrated that LPS induced the expression of STIM1 mRNA and protein in ECs of both human and mouse lungs [44]. Other researchers have pointed out that STIM1 is activated by ROS through the Orai1 channel, leading to an increase in cytosolic calcium (Ca^2+^) in the lungs exposed to LPS. This finding suggests that STIM1/Orai1 signaling is an important mechanism involved in ROS‐mediated EC function and lung edema in response to LPS exposure [35]. The specific mechanism by which STIM1 influences ARDS remains elusive. Therefore, further analysis was conducted, suggesting that STIM1 could regulate ARDS progression through the MAPK pathway. In our ex vivo ALI model induced by LPS‐stimulated macrophages, we observed that the upregulation of STIM1 was accompanied by enhanced MAPK phosphorylation. This finding supports the notion that STIM1 may serve as a potential initiator or amplifier of MAPK‐driven inflammatory responses. The MAPK pathway, initially known as the ERK pathway, plays a crucial role in regulating gene expression in eukaryotic cells [45]. This signaling pathway controls cellular processes, such as cell growth, proliferation, differentiation, and migration [46]. MAPK pathway is involved in the pathogenesis of ARDS [47]. RDN inhibits the production of inflammatory cytokines and the formation of NETs via downregulating MAPK pathway, thus alleviating LPS‐induced acute lung injury [48]. Consistently, studies in human cells have shown that silencing STIM1 markedly suppresses ATP‐induced phosphorylation of ERK and p38 [49], while in HUVECs, increased STIM1 expression correlates with elevated phosphorylation of Raf, ERK, and p38 [50]; these effects are attenuated upon STIM1 inhibition. Collectively, these findings indicate that STIM1 regulates MAPK activity upstream via the Ca^**2**+^/SOCE pathway. Therefore, we propose that in acute lung injury (ALI), STIM1 acts as an upstream regulatory molecule playing a pivotal role in modulating MAPK activation. This observation provides a functional bridge linking our clinical and bioinformatic findings with the underlying cellular mechanisms. These results suggest that STIM1 may regulate MAPK in ARDS; the precise underlying mechanism, however, remains to be further investigated.

## 5. Conclusion

In conclusion, among ICU‐admitted ARDS patients receiving mechanical ventilation, blood and BALF STIM1 mRNA levels were significantly associated with ARDS severity and could predict the risk of 28‐day mortality. Elevated STIM1 mRNA level was found to be correlated with increased disease severity and higher mortality risk. When incorporated into predictive models, STIM1 demonstrated strong potential for assessing both mortality risk and ARDS severity. The findings above indicated that STIM1 mRNA could serve as a novel biomarker for identifying high‐risk ARDS patients, enabling early clinical interventions to improve patient outcomes.

## Ethics Statement

The research involving human subjects was ethically approved by the Ethics Committee of WuHan Third Hospital and conducted in compliance with local laws and institutional guidelines. Written informed consent was obtained from all participants prior to their involvement in the study.

## Disclosure

All authors reviewed the manuscript. S.D., Q.W., D.Z., and L.W. contributed equally to this work.

## Conflicts of Interest

The authors declare no conflicts of interest.

## Author Contributions

S.D.: writing, validation, analysis, drawing. Q.W.: visualization, validation. F.D.: methodology, funding acquisition, software. L.W.: visualization, validation. F.N.: investigation. J.L.: validation, methodology, investigation, supervision, formal analysis.

## Funding

This study is supported by the Hubei Science Foundation project, no. 2024AFB1031, and the Hubei Province Health and Health Commission Clinical Medical Education Teaching Reform Research (Western Medicine) Project, no. HBJG‐250056.

## Supporting Information

Additional supporting information can be found online in the Supporting Information section.

## Supporting information


**Supporting Information 1** Figure S1: Full‐length membrane of the Western blot experiment conducted in this study.

**Supporting Information 2** Figure S2: Identification and functional enrichment analysis of differentially expressed genes (DEGs) between the STIM1‐high and STIM1‐low groups.

**Supporting Information 3** Figure S3: Construction of a protein‐protein interaction (PPI) network of genes.

## Data Availability

The data that support the findings of this study are available from the corresponding author upon reasonable request.
