# Supplementary material for: STIM1 as an Early Predictive Biomarker for Acute Respiratory Distress Syndrome (ARDS) and Its Potential Mechanisms
Source: Hum Mutat. 2026 Apr 17;2026:9013000. doi: 10.1155/humu/9013000 (PMC13090534; doi:10.1155/humu/9013000)

Figure 2

1.β-actin

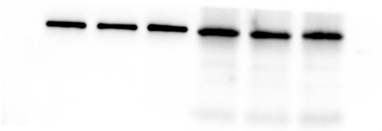

2.STIM1

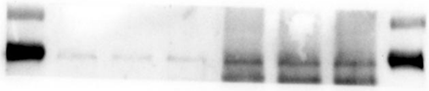

Figure 8

1.β-actin

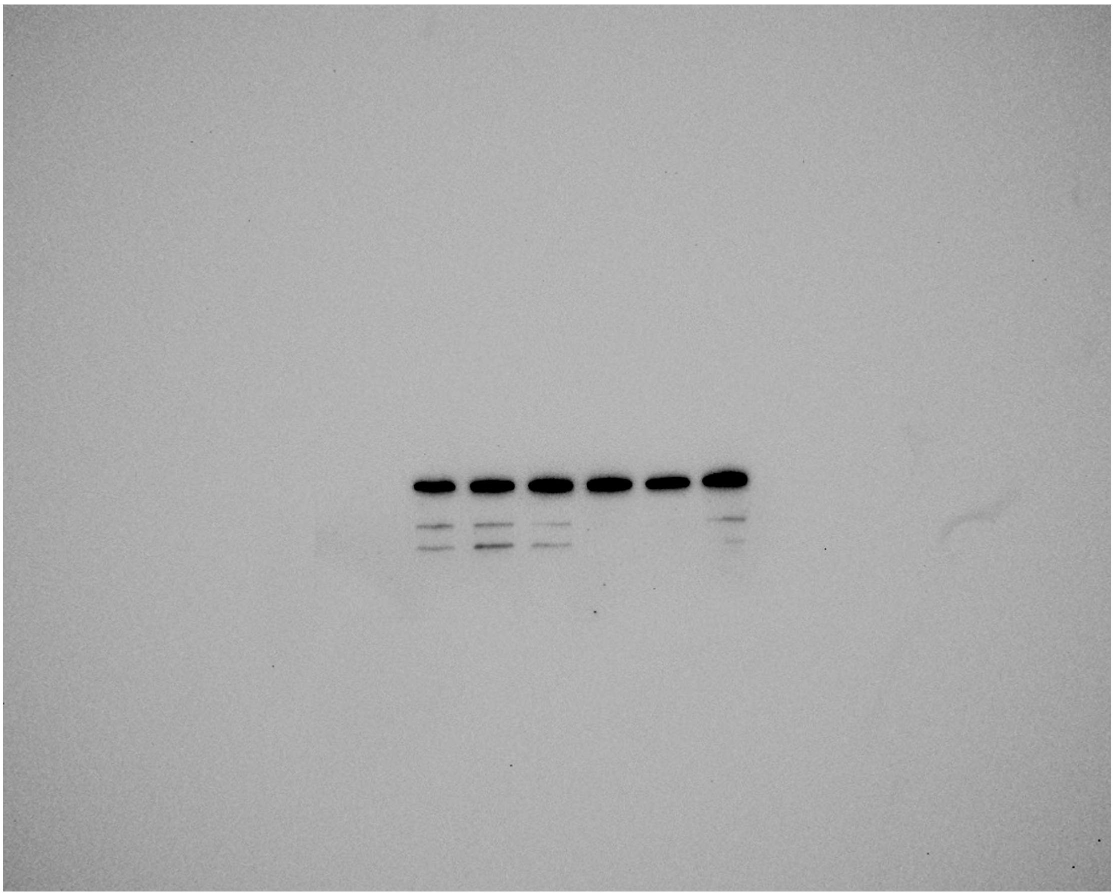

2.p38

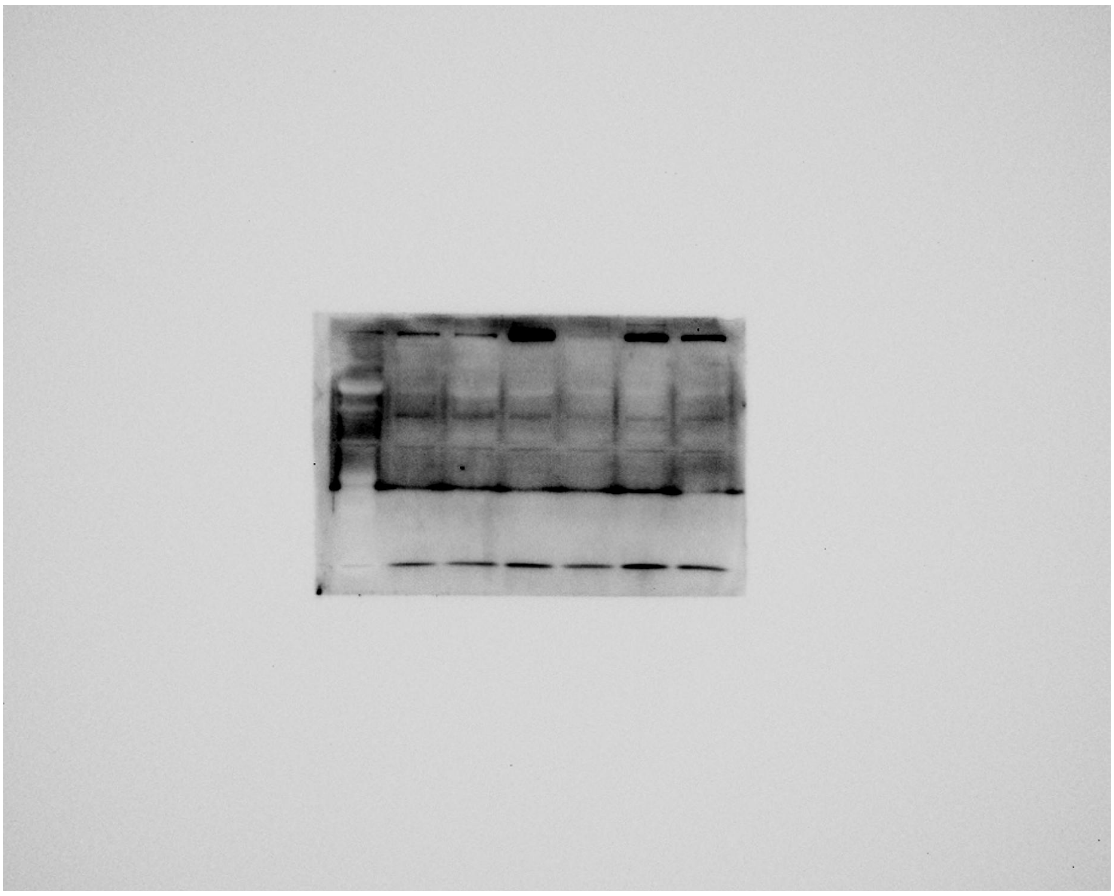

3.p-p38

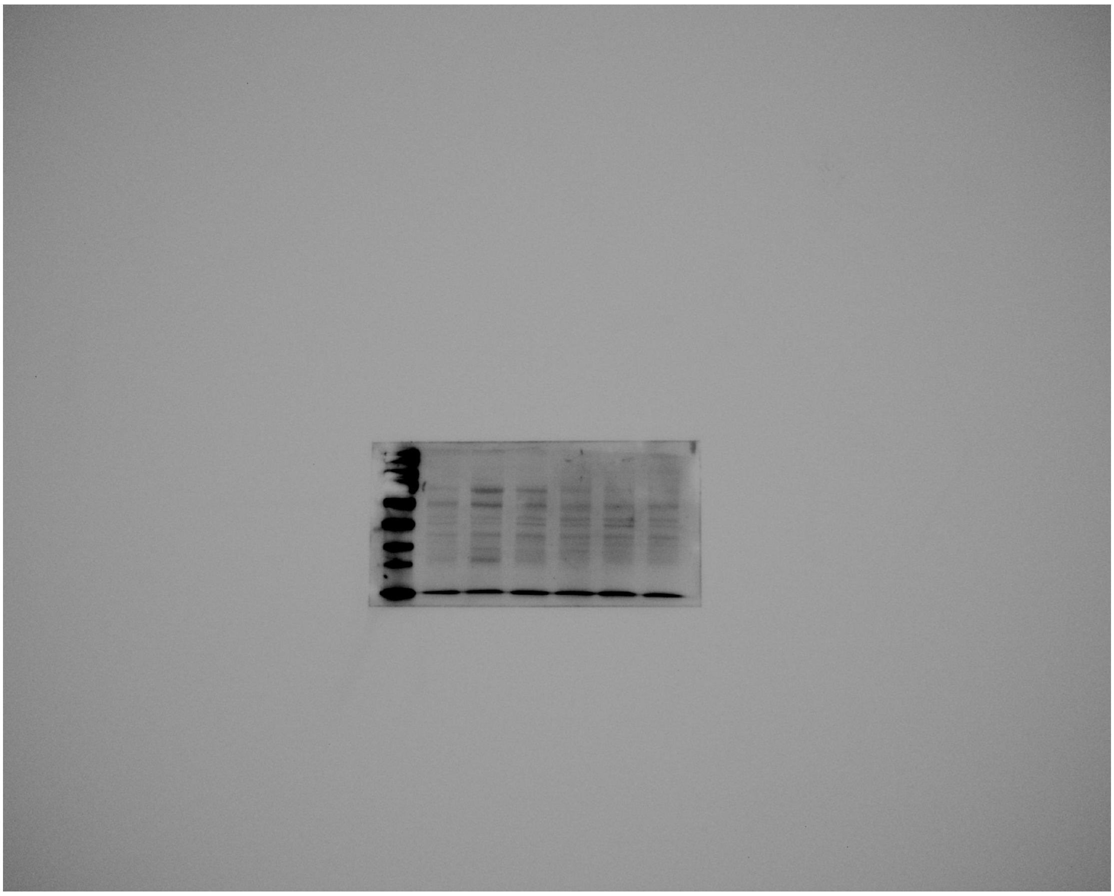

4.ERK

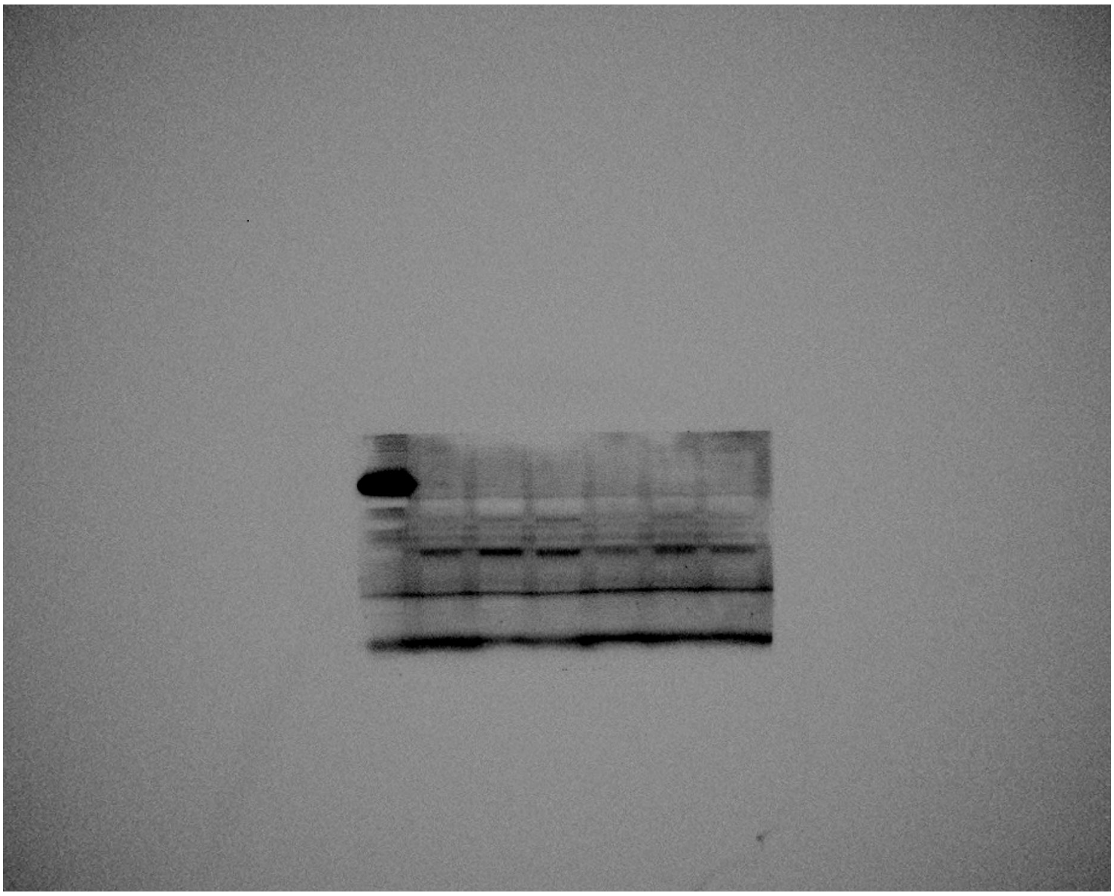

5.p-ERK

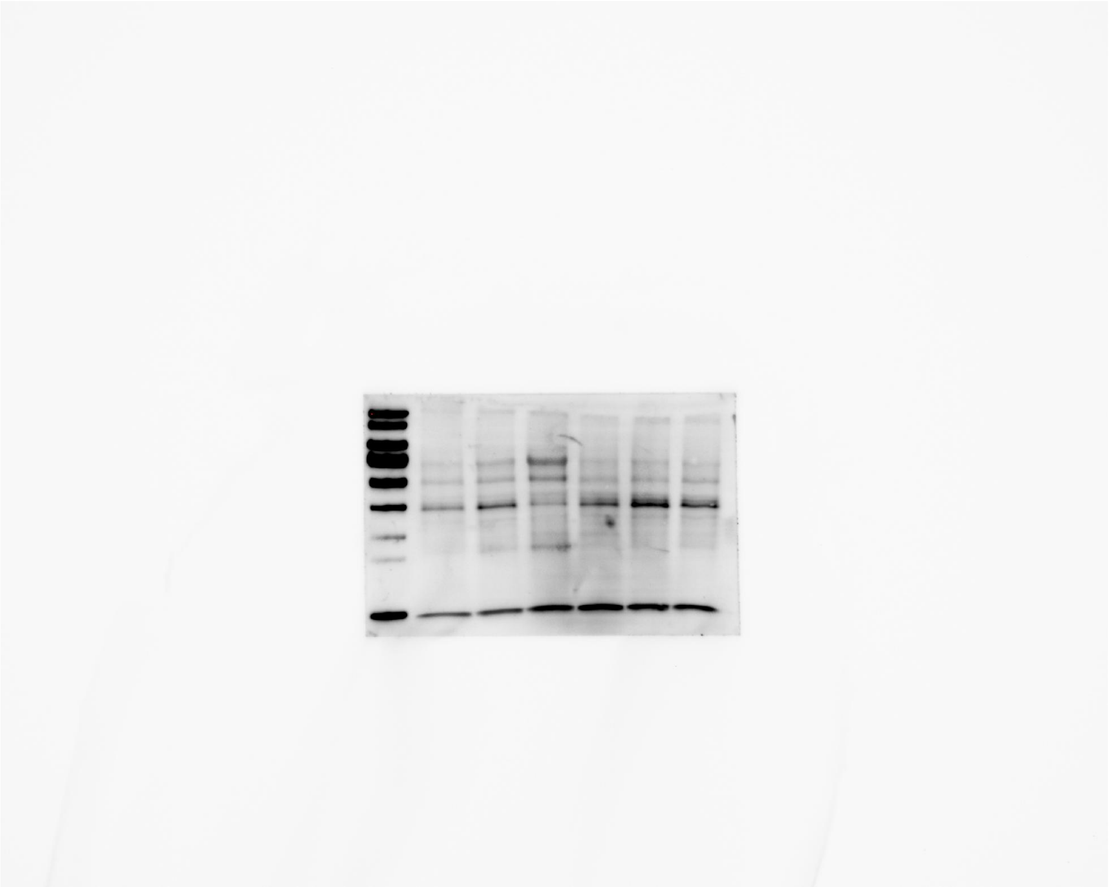

6.JNK

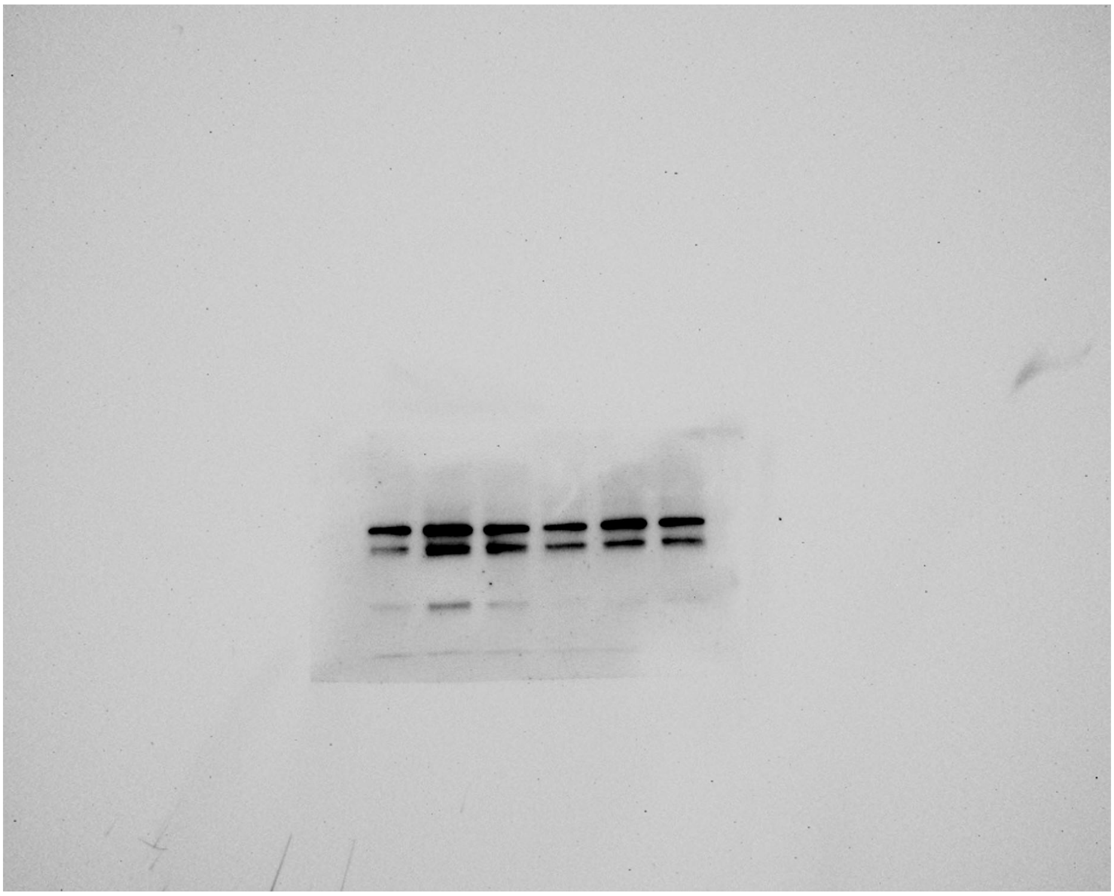

7.p-JNK

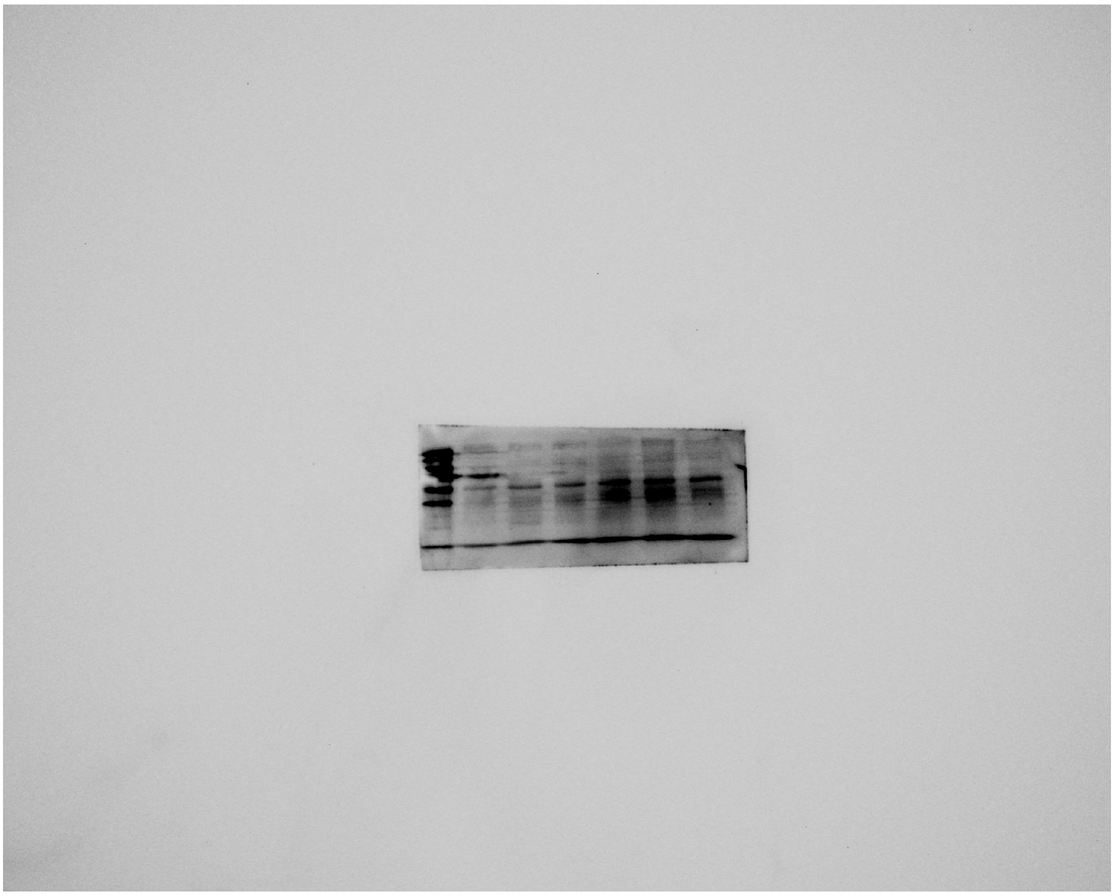

Figure9

1.β-actin

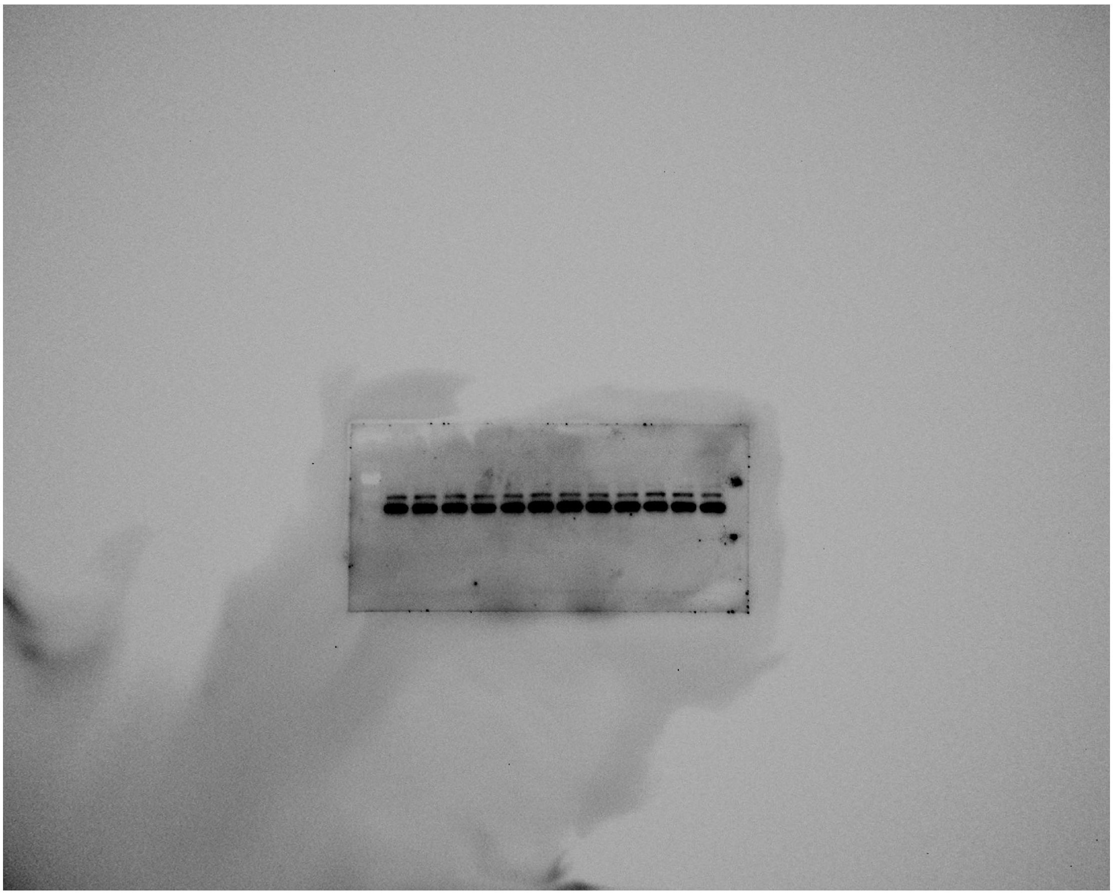

2.STIM1

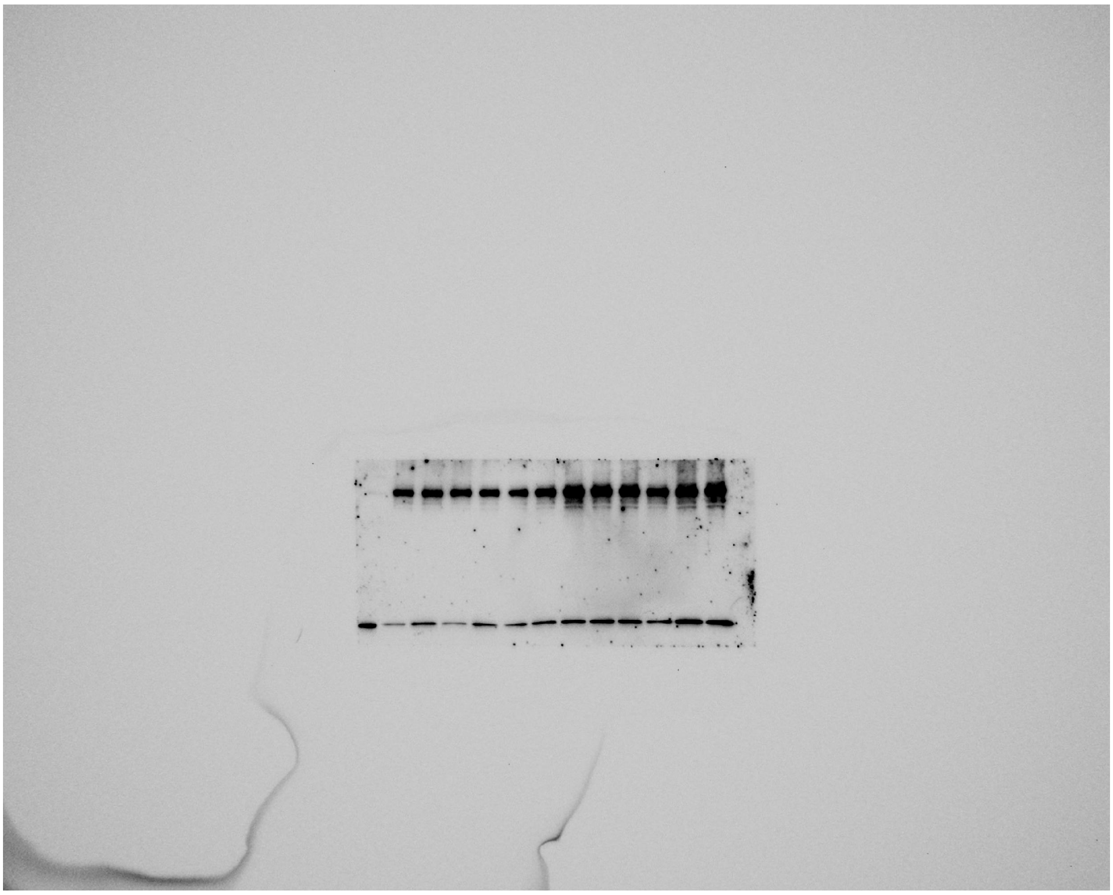

3.p38

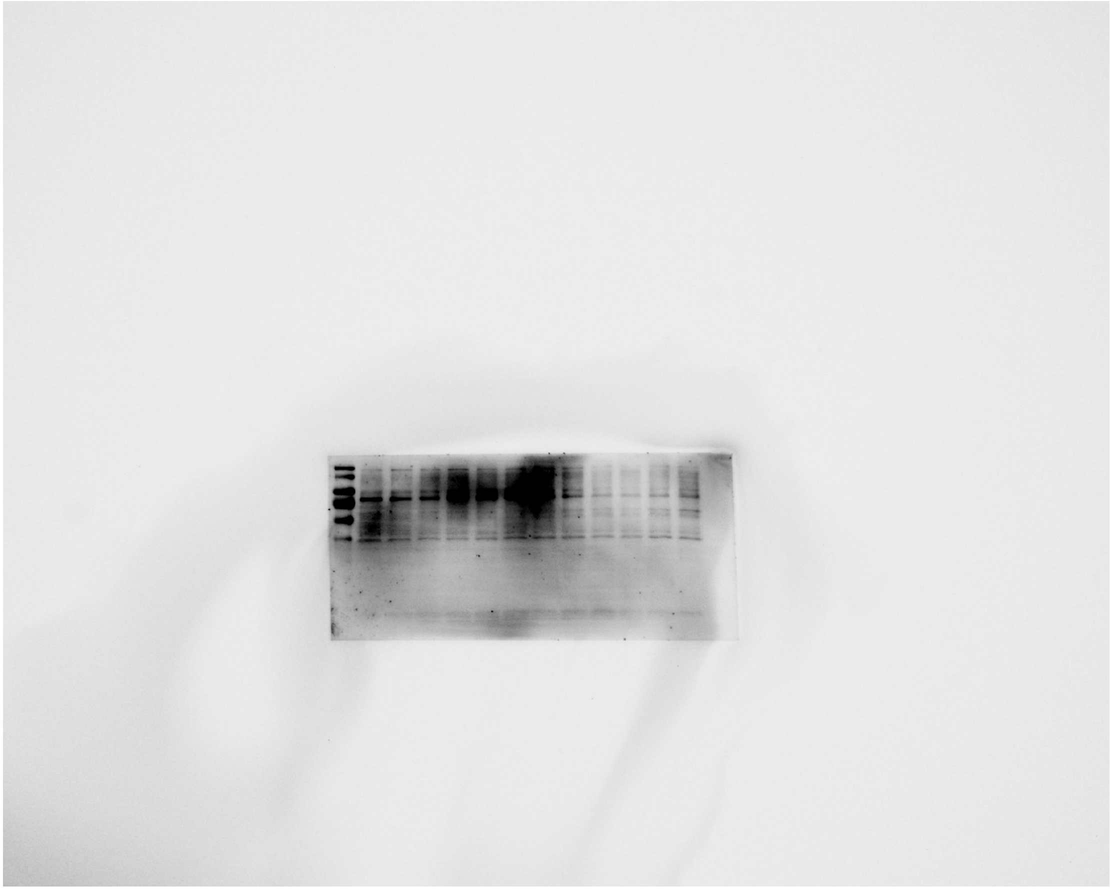

4.p-p38

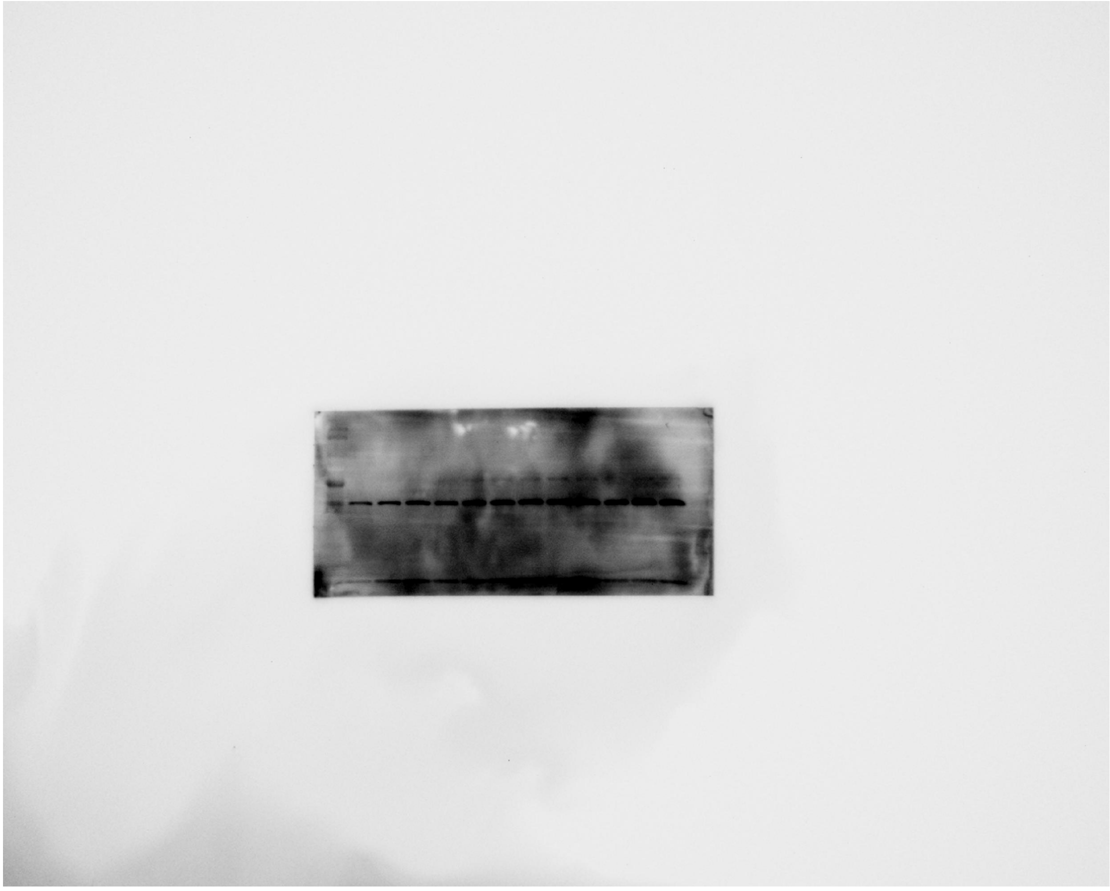

5.ERK

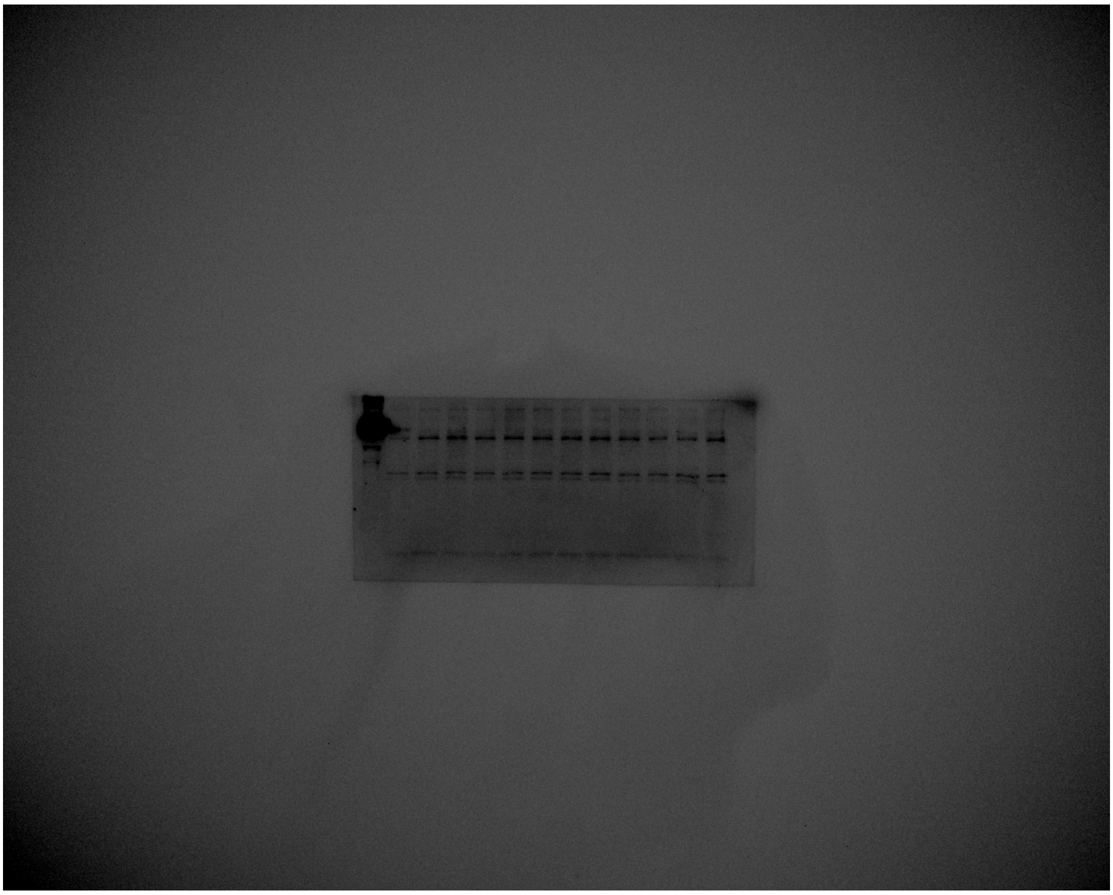

6.p-ERK

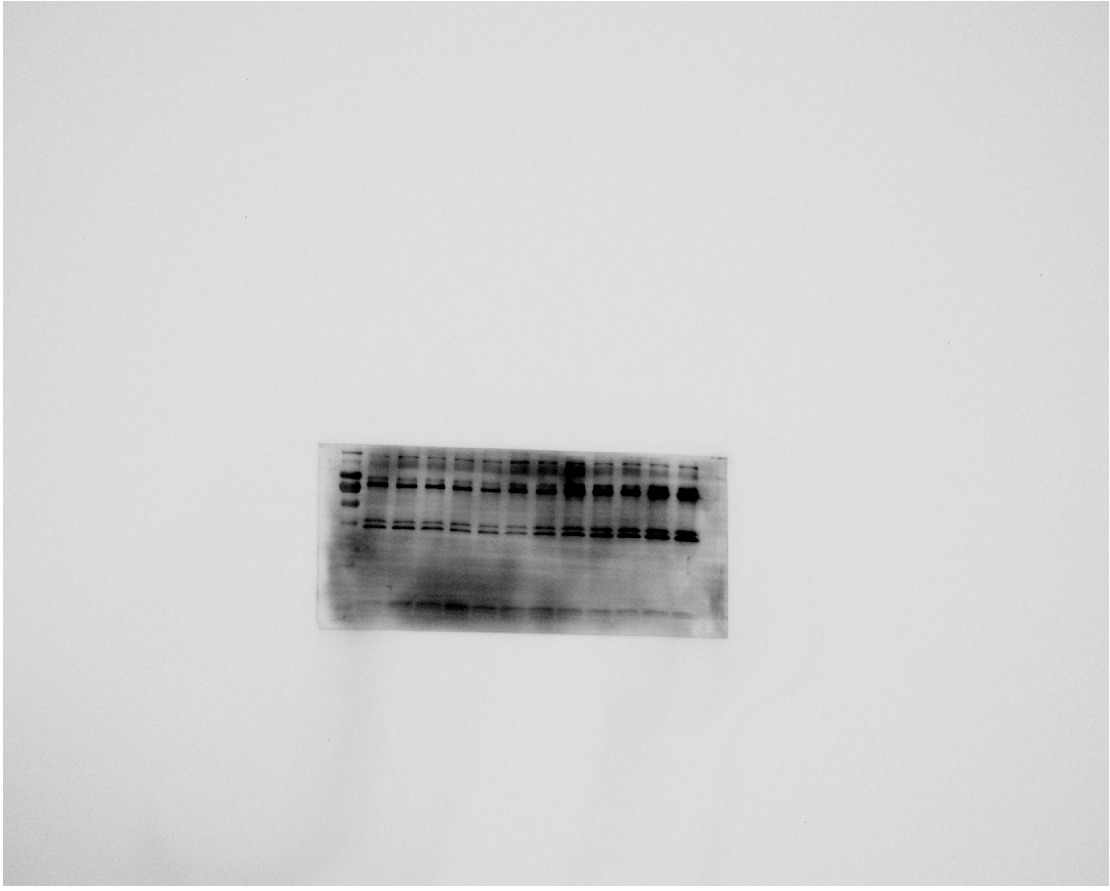

7.JNK

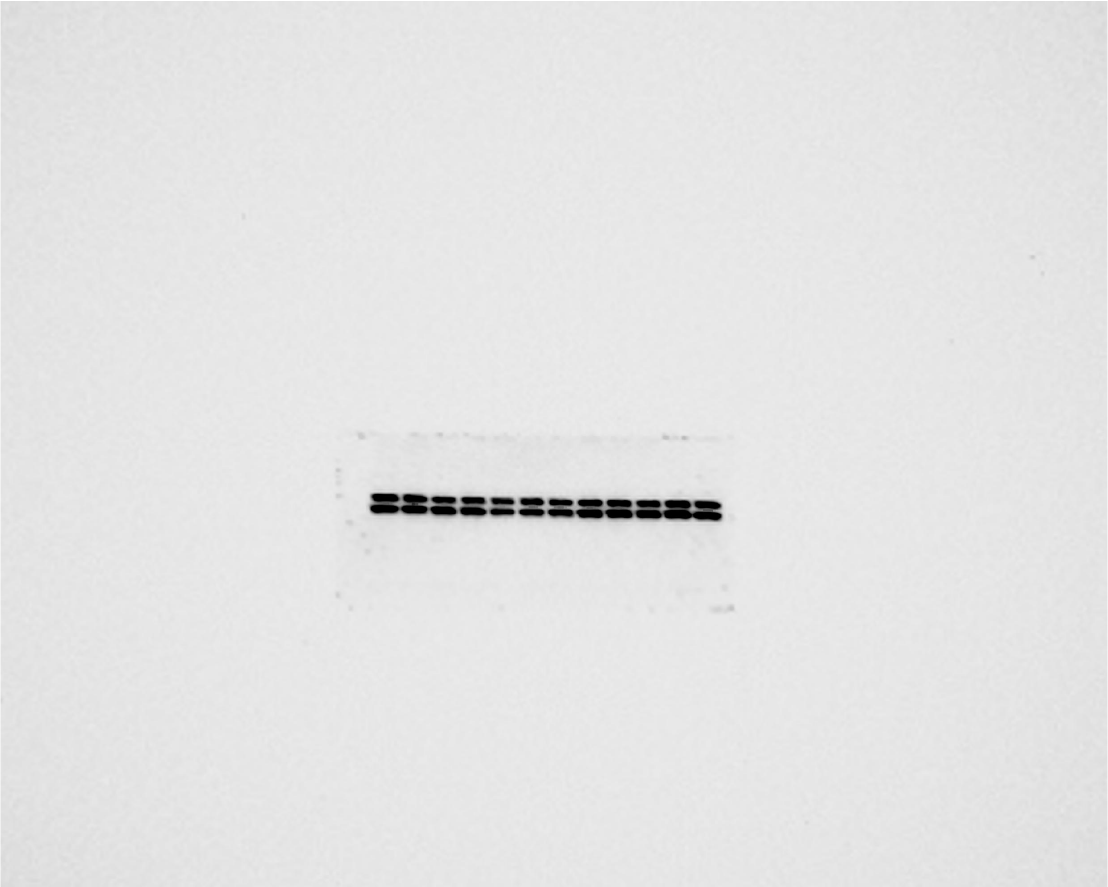

8.p-JNK

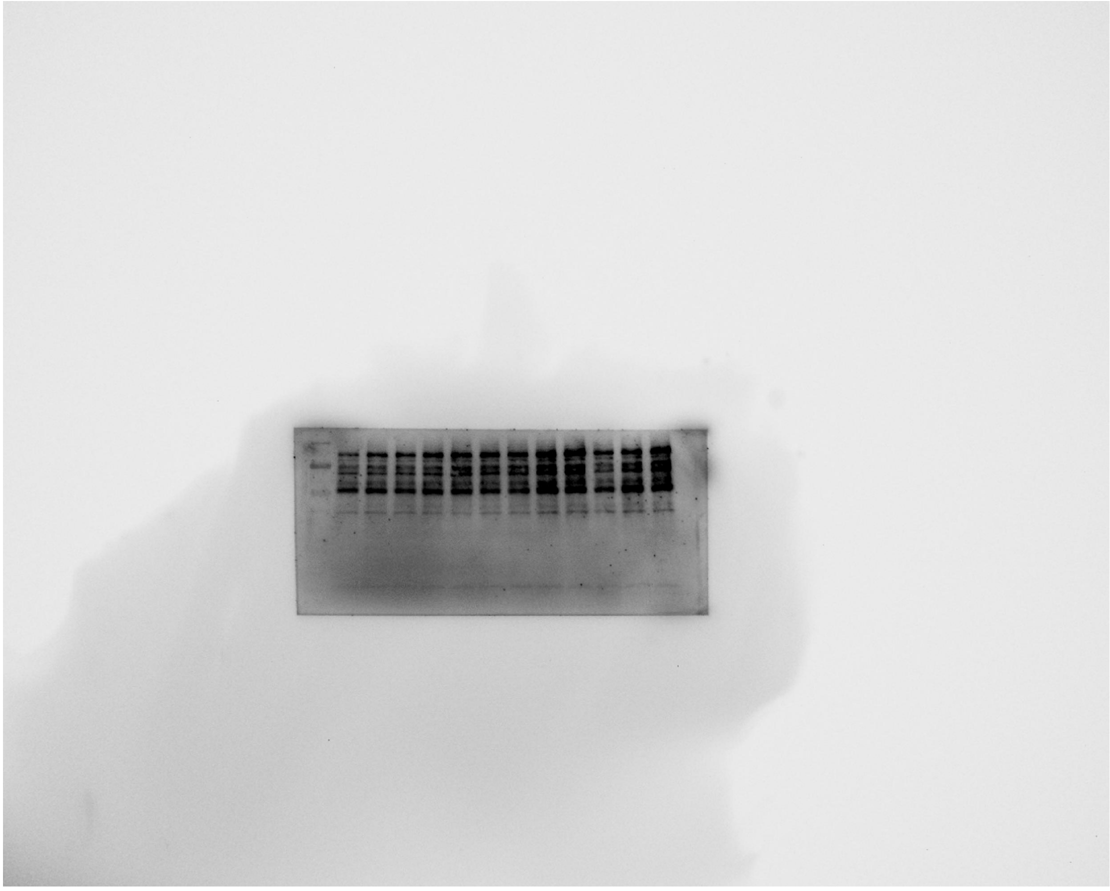

Supplement: Supplementary file 1 — Supporting Information 1 Figure S1: Full‐length membrane of the Western blot experiment conducted in this study. [file HUMU-2026-9013000-s003.pdf]
