# Supplementary material for: STIM1 as an Early Predictive Biomarker for Acute Respiratory Distress Syndrome (ARDS) and Its Potential Mechanisms
Source: Hum Mutat. 2026 Apr 17;2026:9013000. doi: 10.1155/humu/9013000 (PMC13090534; doi:10.1155/humu/9013000)
Supplement: Supplementary file 2 — Supporting Information 2 Figure S2: Identification and functional enrichment analysis of differentially expressed genes (DEGs) between the STIM1‐high and STIM1‐low groups. [file HUMU-2026-9013000-s002.pdf]

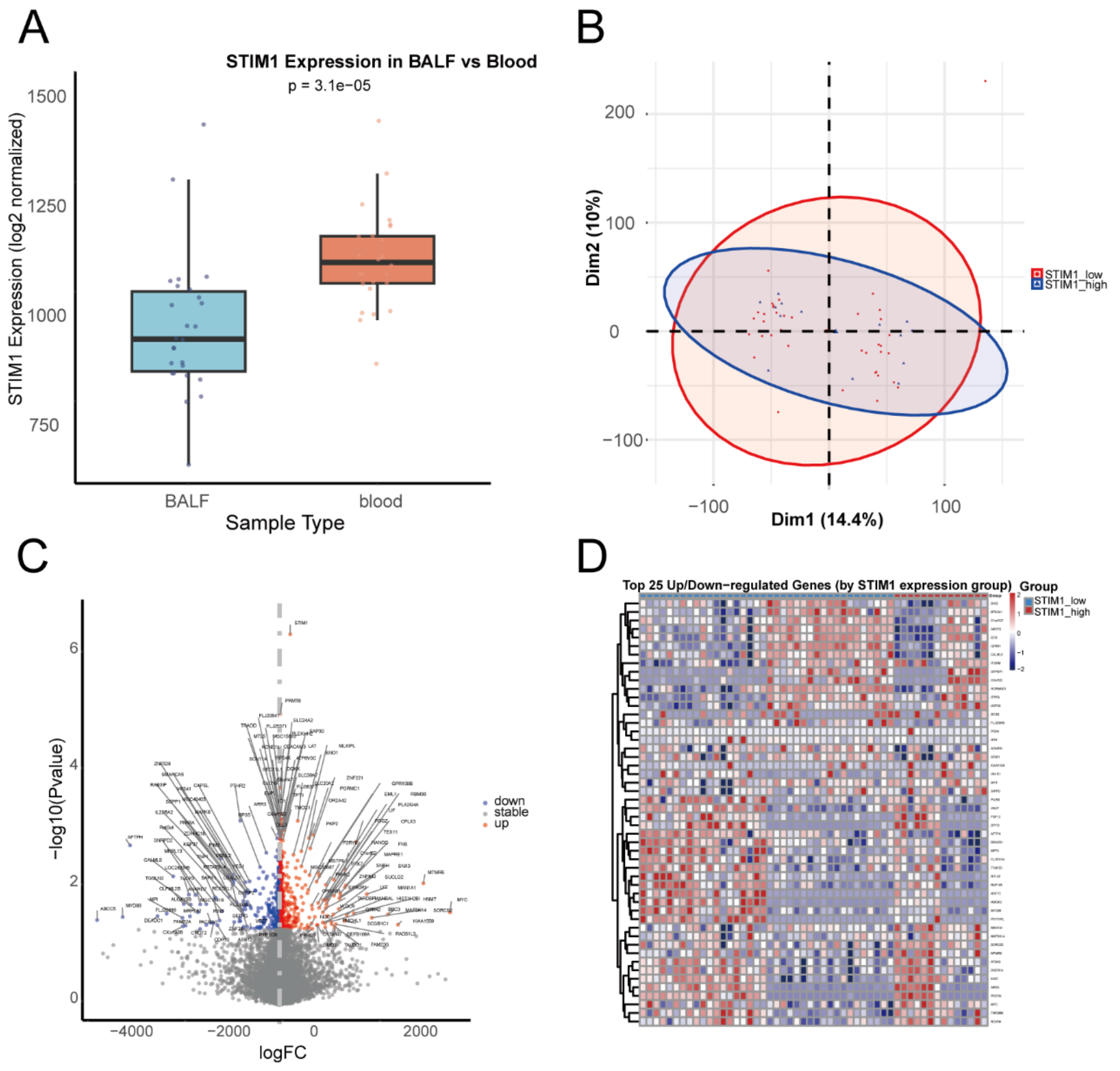

Supplementary FigureS2. Identification of DEGs between the STIM1-high and STIM1-low groups and functional enrichment analysis.

A. STIM1 mRNA expression levels in BALF and blood in ARDS
B. PCA of the ARDS dataset; the ARDS group is separated from other groups. Dim1 and Dim2 are the principle components 1 and 2 generated by PCA; the distribution plot presents the distribution of different group of sample.
C. Volcano plot shows the DEGs by comparing STIM1-high and STIM1-low group from the GSE89953 dataset; red dot represents upregulated DEGs; blue dot represents downregulated DEGs; gray dot represents no significantly DEGs.
D. heatmap of the 50 most significant DEGs according to the adjusted p value; blue indicates downregulated genes; red indicates upregulated genes.
