# Supplementary figures and images for: STIM1 as an Early Predictive Biomarker for Acute Respiratory Distress Syndrome (ARDS) and Its Potential Mechanisms
Source: Hum Mutat. 2026 Apr 17;2026:9013000. doi: 10.1155/humu/9013000 (PMC13090534; doi:10.1155/humu/9013000)

**Supplementary Fiugure S3**

**
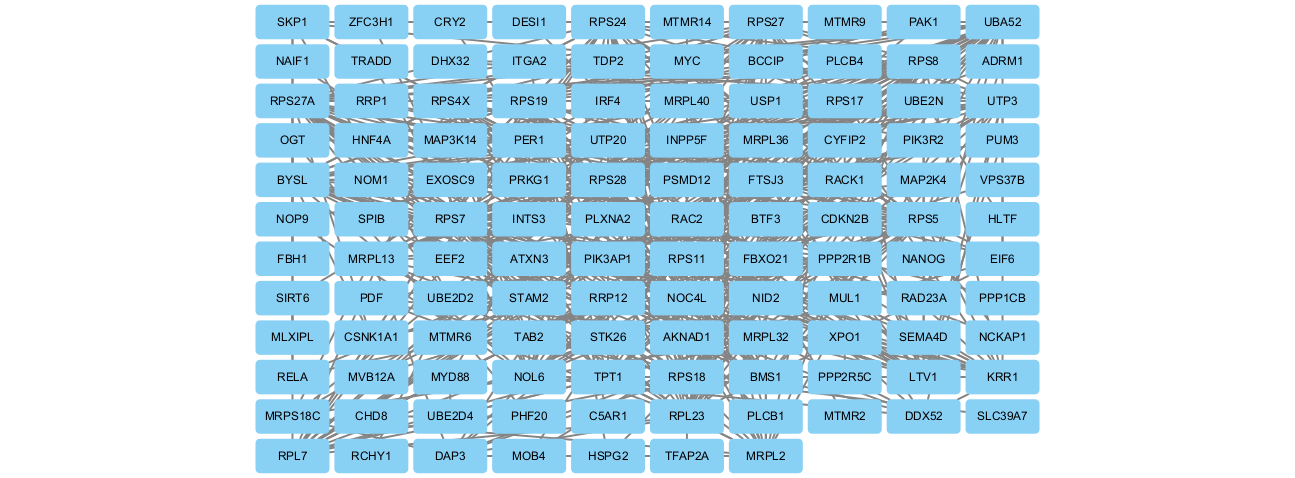
**

Supplementary Figure S3: Construction of a PPI network of genes.

Supplement: Supplementary file 3 — Supporting Information 3 Figure S3: Construction of a protein‐protein interaction (PPI) network of genes. [file HUMU-2026-9013000-s001.doc]
